# Optimization of Ultrasonic Extraction Parameters for the Recovery of Phenolic Compounds in Brown Seaweed: Comparison with Conventional Techniques

**DOI:** 10.3390/antiox13040409

**Published:** 2024-03-28

**Authors:** Zu Jia Lee, Cundong Xie, Xinyu Duan, Ken Ng, Hafiz A. R. Suleria

**Affiliations:** School of Agriculture, Food and Ecosystem Sciences, Faculty of Science, The University of Melbourne, Parkville 3052, Australia; zujial@student.unimelb.edu.au (Z.J.L.); cundongx@student.unimelb.edu.au (C.X.); xiduan@student.unimelb.edu.au (X.D.); ngkf@unimelb.edu.au (K.N.)

**Keywords:** antioxidant capacity, response surface methodology, Australian seaweed, bioactive compounds, HPLC-PDA, LC-ESI-QTOF-MS/MS

## Abstract

Seaweed, in particular, brown seaweed, has gained research interest in the past few years due to its distinctive phenolic profile that has a multitude of bioactive properties. In order to obtain the maximum extraction efficiency of brown seaweed phenolic compounds, Response Surface Methodology was utilized to optimize the ultrasound-assisted extraction (UAE) conditions such as the amplitude, time, solvent:solid ratio, and NaOH concentration. Under optimal conditions, UAE had a higher extraction efficiency of free and bound phenolic compounds compared to conventional extraction (stirred 16 h at 4 °C). This led to higher antioxidant activity in the seaweed extract obtained under UAE conditions. The profiling of phenolic compounds using LC-ESI-QTOF-MS/MS identified a total of 25 phenolics with more phenolics extracted from the free phenolic extraction compared to the bound phenolic extracts. Among them, peonidin 3-*O*-diglucodise-5-*O*-glucoside and hesperidin 5,7-*O*-diglucuronide are unique compounds that were identified in *P. comosa*, *E. radiata* and *D. potatorum,* which are not reported in plants. Overall, our findings provided optimal phenolic extraction from brown seaweed for research into employing brown seaweed as a functional food.

## 1. Introduction

At present, the innovation and increased sustainability of the food industry calls for diversification of food supply to combat the global food security issue. Thus, considerable attention and effort have been focused on seaweed, an underexploited and sustainable marine crop. Marine seaweed has been widely consumed by the East Asian population for centuries, dating back to 2700 BC in China [1]. With the emergence of epidemiological studies highlighting a causal association between reduced risk of metabolic diseases and seaweed consumption, the consumption of seaweed in Western diet has steadily increased over the past decade [2,3,4,5]. There is a total of 145 edible seaweed species cultured or wildly harvested globally (mainly in East and Southeast Asia) which include green seaweed (20%), brown seaweed (26%), and red seaweed (54%) [6]. Australia’s coastal water harbours a high number of endemic seaweed species, many of which have immense untapped potentials for food and nutraceutical applications [7].

Among these seaweeds, brown seaweed has emerged as a high biotechnological potential marine crop due to its numerous bioactive compounds which confer to multiple health benefits [8,9]. Brown seaweed contains high phenolic content and presence of phlorotannin which are not found in land plants [10]. From a chemistry perspective, phenolic compounds are characterized by the reducing hydroxyl group(s) on a benzene ring which can be by itself or a component of a heterocyclic ring compound. The unique structure of some phenolic compounds (phenolic acids and polyphenols) gives rise to strong antioxidant activity in scavenging free radicals and reactive oxygen species, in addition to chelating oxidative metal ions and inhibiting oxidative enzyme activities [11]. These phenolic antioxidant activities endow many useful features that are related to further oxidative related bioactivities such as anti-inflammatory, antidiabetic, and anti-neurodegenerative properties [11].

Conventionally, the extraction of phenolics from seaweeds is conducted with organic solvents stirred overnight, but the yield is low. Thus, new technologies have since been developed to extract seaweed phenolics more efficiently. One of which is by utilizing ultrasound-assisted extraction (UAE), which has gained popularity over conventional extraction method due to its shorter extraction time, lower amount of solvent used, and increased extraction yield and phenolic quality [11,12]. Ultrasonication is a process whereby ultrasonic waves are introduced to produce acoustic cavitations in the extraction solvent to form cavitation bubbles [13]. This introduces a mechanical effect which disrupts the algae cell wall and enhances the mass transfer of phenolics into the extraction solvent [13]. The cell wall matrix of seaweed differs from that of land plants in terms of their chemical and structural composition [14]. Hence, the processing condition of ultrasonication for seaweed phenolic extraction could vary from that of land plants. 

In this study, we assessed the phenolic composition and antioxidant capacity of five brown seaweed species, aiming to unlock the potential value of these seaweed phenolics for nutraceutical, nutritional, and pharmacological uses. For these purposes, we developed and optimized an alternative method based on UAE for the recovery of phenolic compounds from brown seaweed. Response surface methodology (RSM) is a collection of statistical and mathematical techniques that evaluate the effect of several processing parameters and their interaction as well as develop, improve, and optimize these parameters. UAE parameters such as the solvent:solid ratio, time, amplitude, and NaOH concentration were optimized using RSM, by employing a three-level, three-variable Box–Behnken Design (BBD) to obtain the optimal condition for the extraction of brown seaweed phenolics from five collected samples. To obtain a more comprehensive knowledge of the extracted brown seaweed phenolics, high-performance liquid chromatography coupled with a photodiode array detector (HPLC-PDA) and liquid chromatography electrospray ionization quadrupole time-of-flight mass spectrometry (LC-ESI-QTOF-MS/MS) was utilized to determine the phenolic composition of these brown seaweeds.

## 2. Materials and Methods

### 2.1. Chemicals and Materials

Organic solvents used for extraction were purchased from Sigma Aldrich (Castle Hill, NSW, Australia). Other chemicals and standards of analytical grade or higher were also sourced from Sigma Aldrich, such as 2,2′-diphenyl-1-picrylhydrazyl (DPPH), 2′-azino-di-(3-ethylbenzthiazoline sulfonic acid) (ABTS), 3-(2-Pyridyl)-5,4,4-dimethoxybenzaldehyde,4-triazine-*p*,5,6-diphenyl-1,6-hydroxy-2,6-Tris(2-pyridyl)-s-triazine (TPTZ), 7,8-tetramethylchroman-2-carboxylic acid (Trolox), aluminium trichloride, anhydrous sodium carbonate, catechin hydrate, disodium ethylenediaminetetraacetic acid (EDTA-Na_2_), ferrous chloride, Folin–Ciocalteu reagent, gallic acid monohydrate, phloroglucinol, potassium persulfate, *p*’-disulfonic acid monosodium salt hydrate (Ferrozine), sodium acetate, trisodium phosphate, and quercetin. HPLC grade standards including phloroglucinol, gallic acid, chlorogenic acid, syringic acid, synaptic acid, catechin, epicatechin, and epigallocatechin were purchased from Sigma Aldrich (Castle Hill, NSW, Australia). Vanillin was obtained from Chem-Supply Pty Let., Adelaide, SA, Australia. The Milli-Q water used was obtained from Millipore Milli-Q Gradient Water Purification System (Darmstadt, Germany). 

### 2.2. Seaweed Sample Preparation

Five brown seaweed samples (*Cytospora* sp., *Durvillaea potatorum*, *Sargassum fallax*, *Ecklonia radiata*, and *Phyllospora comosa*) were collected from Australia’s coastal region (38°15′54.0″ S 144°40′10.3″ E) during Spring 2023. The seaweed samples were washed under running water to remove traces of sand, sediments, and other impurities. Following this, the seaweed samples were freeze-dried (Dynavac designed FD3, Hingham, MA, USA) at −50 °C for 72 h. The freeze-dried samples were grounded into powder using a grinder (Cuisinart^®^ Spice and Nut Grinder, SG-10A, Asquith, NSW, Australia) and subsequently stored at −20 °C for further extraction and analysis. 

### 2.3. Conventional Extraction of Free and Bound Phenolics 

Conventional extraction of free phenolic compounds was carried out according to the method by Subbiah et al. [15]. The freeze-dried seaweed samples were extracted using 70% ethanol with 0.1% formic acid in a shaking incubator (ZWYR-240 incubator shaker, Labwit, Ashwood, VIC, Australia) at 120 rpm, 4 °C for 16 h at solvent to seaweed ratio of 10:1 (mL:g). After the extraction, the samples were centrifuged at 8000 rpm for 15 min at 4 °C using a Hettick Refrigerated Centrifuge (ROTINA380R, Tuttlingen, Baden-Württemberg, Germany). The supernatants were then collected as conventional free phenolic extract fraction. The residuals were then washed with 70% ethanol thrice and then air-dried in the fumehood for three days.

Bound phenolics were extracted from the washed and dried residue using a modified version of the alkaline hydrolysis method [16]. Briefly, 1 g of the dried residue was treated with 10 mL of 2M NaOH solution in a shaking incubator (ZWYR-240 incubator shaker, Labwit, Ashwood, VIC, Australia) at 37 °C for 1 h. The mixture was neutralized with 2N HCl, followed by the addition of 10 mL of 70% ethanol with 0.1% formic acid. The mixture was then incubated again at 4 °C for 16 h in the shaking incubator (ZWYR-240 incubator shaker, Labwit, Ashwood, VIC, Australia) at 120 rpm. The mixture was centrifuged at 8000 rpm and 4 °C for 15 min and the supernatant was collected as the bound phenolic extracts fraction. Both phenolic fractions were stored at −20 °C for further analysis.

### 2.4. Ultrasonic Extraction of Free and Bound Phenolics

For ultrasonic extraction, 1 g of freeze-dried seaweed sample was added with 70% ethanol with 0.1% formic acid, and different samples similarly obtained were ultrasonicated at different amplitude, time, and solid:solvent ratio using a Branson Digital Sonifier (102C, Danbury, CT, USA) according to Table 1. The free phenolic fraction (supernatant) was collected after centrifugating at 8000 rpm and 4 °C for 15 min using Hettich Refrigerated Centrifuge (ROTINA380R, Tuttlingen, Baden-Württemberg, Germany). All free phenolic fractions were stored at −20 °C for further analysis. The optimized ultrasonic extraction parameters were determined by using the total phenolic content (TPC), total phlorotannin content (TPhC), and antioxidant capacity (DPPH radical scavenging activity) of the extracts.

The residues were washed with 70% ethanol thrice and air-dried in the fumehood for three days. Following this, bound phenolics were extracted from the washed residues as follows: 10 mL of NaOH solution were added to 1 g of dried residue and ultrasonicated at different amplitudes, times, and NaOH concentrations in Branson Digital Sonifier (102C, Danbury, CT, USA) according to Table 1. The mixtures were neutralized with 2N HCl and 10 mL of solvent was added. The mixture was centrifuged at 8000 rpm and 4 °C for 15 min and the supernatants were collected as the bound phenolic fractions and were stored at −20 °C for further analysis. The optimized ultrasonic extraction parameters were determined by analysis of total phenolic content (TPC), total phlorotannin content (TPhC), and antioxidant capacity (DPPH radical scavenging) of the extracts.

### 2.5. Experimental Design

Response Surface Methodology (RSM) using BBD with three factors (X_1_, X_2_, X_3_) at three levels (−1, 0, +1) was generated using DesignExpert Software (Version 12, Stat-Ease, Inc., Minneapolis, MN, USA) to optimize the ultrasonic extraction condition for free and bound phenolics. The investigated independent variables and response values for free and bound phenolic extraction are shown in Table 1. The designs of experiments consisting of 17 trials, comprising 5 repeated tests at the central point for free and bound phenolics, are shown in Tables S1 and S2.

The experimental results were fitted to a 2nd-order polynomial model and the regression coefficient was recorded. The proposed general model for the response surface analysis is as follow:

$$Y_{i}=\beta_{0}+\sum_{n=1}^{n}\beta_{i}X_{i}+\sum_{i=1}^{n}\beta_{ii}X_{i}^{2}\sum_{i=1}^{n}\sum_{j-1+1}^{n}\beta_{ij}X_{i}X_{j}$$

### 2.6. Characterization of Free and Bound Phenolics from Conventional and Ultrasonic Extraction

#### 2.6.1. Determination of Total Phenolic Content (TPC)

The total phenolic content (TPC) of seaweed extracts was determined using a modified method from previous study published by Wu, Gao, Wang, Peng, Guo, Ma, Zhang, Zhang, Wu, and Xiao [16]. Briefly, 25 μL of sample was incubated with 25 μL of Folin aqueous solution (25% *v*/*v*) at 25 °C for 5 min followed by addition of 25 μL of sodium carbonate solution (10% *w*/*w*). The mixture was then incubated in the dark at 25 °C for 1 h. Absorbance of the mixtures were measured at λ765 nm using a Multiskan Go Microplate photometer (Thermo Fisher Scientific, Waltham, MA, USA). Standard calibration curve was constructed using gallic acid (0–200 μg/mL) in ethanol. The results were expressed as mean gallic acid equivalent (GAE) ± standard deviation based on dry weight of seaweed (mg GAE/g ± SD).

#### 2.6.2. Determination of Total Flavonoid Content (TFC)

The total flavonoid content (TFC) of the seaweed extracts was determined according to the method described by Duan et al. [17]. The samples (80 μL) were incubated with 80 μL of aluminum trichloride (2%, *w*/*v*) and 120 μL sodium acetate (50 g/L) at 25 °C for 2.5 h in the dark. Absorbance of the mixtures were measured at λ440 nm. Standard calibration curve was constructed using quercetin (0–50 μg/mL) in ethanol. The results were expressed as mean quercetin equivalent (QE) ± standard deviation based on dry weight of seaweed (mg QE/g ± SD).

#### 2.6.3. Determination of Total Condensed Tannin (TCT)

The total condensed tannins (TCTs) of the seaweed extracts were determined as vanillin reactive substances, as reported by Subbiah, Ebrahimi, Agar, Dunshea, Barrow, and Suleria [15]. Firstly, 25 μL of sample was mixed with 150 μL vanillin (4% *w*/*v*) and 25 μL methanolic sulfuric acid solution (32% *v*/*v*). The mixture was then incubated at 25 °C for 15 min in the dark. Absorbance of the mixtures were measured at λ500 nm. Standard calibration curve was constructed using catechin (0–1000 μg/mL) in methanol. The results were expressed as mean catechin equivalent (CE) ± standard deviation based on dry weight of seaweed (mg CE/g ± SD).

#### 2.6.4. Analysis of Total Phlorotannin Content (TPhC)

The total phlorotannin content of the seaweed extracts was determined using the 2,4-dimethoxybenzaldehyde (DMBA) assay method as described by Subbiah, Ebrahimi, Agar, Dunshea, Barrow, and Suleria [15]. DMBA solution was first prepared by mixing equal volumes of DMBA in acetic acid (2%, *w*/*v*) and hydrochloric acid in acetic acid (6% *v*/*v*). Then, 25 μL sample was incubated with 125 μL of DMBA solution at 25 °C for 60 min in the dark. Absorbance of the mixtures were measured at λ510 nm. Standard calibration curve was constructed using phloroglucinol (0–100 μg/mL) in ethanol. The results were expressed as mean phloroglucinol equivalent (PGE) ± standard deviation based on dry weight of seaweed (mg PGE/g ± SD).

#### 2.6.5. DPPH Radical Scavenging Activity

DPPH radical scavenging activity was determined as described by Ummat et al. [18] with modification. In a 96-well plate, 40 μL of sample was mixed with 260 μL of methanolic DPPH solution (0.1 mM). The mixture was incubated at 25 °C for 30 min in the dark. Absorbance of the mixtures were measured at λ517 nm. Standard calibration curve was constructed using Trolox (0–50 μg/mL) in ethanol. The results were expressed as mean Trolox equivalent (TE) ± standard deviation based on dry weight of seaweed (mg TE/g ± SD).

#### 2.6.6. ABTS Radical Scavenging Activity

ABTS radical scavenging activity was determined as described by Wu, Gao, Wang, Peng, Guo, Ma, Zhang, Zhang, Wu, and Xiao [16] with modification. Firstly, stock ABTS radical solution was prepared using 1.25 mL of ABTS (7 mM) and 25 μL of potassium persulfate (140 mM). This mixture was left to oxidize in the dark to generate the radical for 16 h. Following this, the ABTS radical solution was diluted to ~0.7 absorbance unit at λ734 nm. In a 96-well plate, 10 μL of sample was incubated with 290 μL of the ABTS radical, and the mixture was incubated at 25 °C for 6 min in the dark. Absorbance of the mixtures were measured at λ734 nm. Standard calibration curve was constructed using Trolox (0–200 μg/mL) in ethanol. The results were expressed as mean Trolox equivalent (TE) ± standard deviation based on dry weight of seaweed (mg TE/g ± SD).

#### 2.6.7. Ferric-Reducing Antioxidant Power (FRAP)

The ferric-reducing antioxidant power (FRAP) of the samples were measured according to Ummat, Tiwari, Jaiswal, Condon, Garcia-Vaquero, O’Doherty, O’Donnell, and Rajauria [18] with modification. The FRAP reagent containing the TPTZ-Fe[III] complex was prepared by mixing 25 mL sodium acetate solution (300 mM), adjusted to pH ~3.6 using NaOH, 2.5 mL TPTZ solution (10 mM *w*/*v*, HCL added to boost solubility), and 2.5 mL FeCl_3_ solution (20 mM). Then, 20 μL of sample was incubated with 280 μL of the FRAP reagent. The mixture was left to incubate at 37 °C for 10 min in the dark. Absorbance of the mixtures were measured at λ594 nm. Standard calibration curve was constructed using Trolox (0–100 μg/mL) in methanol. The results were expressed as mean Trolox equivalent (TE) ± standard deviation based on dry weight of seaweed (mg TE/g ± SD).

#### 2.6.8. Phosphomolybdate-Reducing Antioxidant Capacity (PRAC)

Phosphomolybdate-reducing antioxidant capacity (PRAC) was determined according to Subbiah, Ebrahimi, Agar, Dunshea, Barrow, and Suleria [15] with modification. The phosphomolybdate reagent was firstly prepared by mixing 10 mL sulfuric acid (0.6 M) with 10 mL trisodium phosphate solution (28 mM) and 10 mL of ammonium molybdate solution (4 mM). In a 96-well plate, 40 μL of sample was incubated with 260 μL of phosphomolybdate solution at 90 °C for 90 min in the dark. After incubation, the mixture was left to cool at 25 °C for 10 min. Absorbance of the mixtures were measured at λ695 nm. Standard calibration curve was constructed using Trolox (0–200 μg/mL) in ethanol. The results were expressed as mean Trolox equivalent (TE) ± standard deviation based on dry weight of seaweed (mg TE/g ± SD).

#### 2.6.9. Ferrous Ion Chelating Activity (FICA)

Ferrous ion chelating activity (FICA) was determined according to Subbiah, Ebrahimi, Agar, Dunshea, Barrow, and Suleria [15] with modification. In brief, 15 μL of sample was mixed with 85 μL water, 50 μL ferrous chloride (2 mM), and 50 μL ferrozine (5 mM). The mixture was left to incubate at 25 °C for 10 min in the dark. Absorbance of the mixtures were measured at λ562 nm. Standard calibration curve was constructed using EDTA (0–50 μg/mL) in ethanol. The results were expressed as mean EDTA equivalent (EDTA-E) ± standard deviation based on dry weight of seaweed (mg EDTA-E/g ± SD).

### 2.7. Quantification of Phenolic Compounds by HPLC-PDA

Phenolic compounds in free and bound extracts of brown seaweeds were quantified using Agilent 1200 series HPLC (Agilent Technologies, Santa Clara, CA, USA) equipped with photo diode array (PDA) according to the method by [19] with some modification. The column used was a Synergi Hydro-Reverse Phase 80 Å, LC column 250 × 4.6 mm, 4 µm (Phenomenex, Torrance, CA, USA). The mobile phase used was (A) 0.25% aqueous formic acid and (B) acetonitrile/water (80/20; *v*/*v*) with 0.25% formic acid with flow rate of 0.5 mL/min and column temperature at 25 °C. The injection volume was constant at 10 μL for samples and standard compounds. The elution conditions applied are as follow: 0–40 min linear gradient from 0–10% B; 40–60 min linear gradient from 10% to 15% of B; 60–80 min linear gradient from 15% to 20% B; 80–90 min linear gradient from 20–30% B; 90–100 min linear gradient from 30–10% B; and finally, washing and conditioning of the column. Absorbance measures were recorded at 254 nm, 280 nm, and 320 nm.

### 2.8. Characterization of Phenolic Compounds by LC-ESI-QTOF-MS/MS Analysis

Extensive characterization of free and bound phenolic compounds from the extracts were carried out using liquid chromatography electrospray ionization quadrupole time-of-flight mass spectrometry (LC-ESI-QTOF-MS/MS) analysis utilizing an Agilent 1200 series HPLC system equipped with an Agilent 6520 Accurate-Mass Q-TOF LC-MS (Agilent Technologies) with an electrospray ionization (ESI) source according to the method described by Duan, Subbiah, Xie, Agar, Barrow, Dunshea, and Suleria [17]. HPLC buffers (Mobile phase A: 100% MilliQ water with 0.1% formic acid, Mobile phase B: acetonitrile/MilliQ water/formic Acid (95:5:0.1)) were firstly deaerated by sonication in an Ultrasonic water bath (Power sonic 505, Gyeonggi-do, Republic of Korea) at 25 °C for 10 min. The separation process was conducted using a Synergi Hydro-Reverse Phase 80 Å, LC column 250 × 4.6 mm, 4 µm (Phenomenex, Torrance, CA, 202 USA) with column temperature set at 25 °C. The sample injection volume was 6 µL. The mobile phase was applied at a flow rate of 0.8 mL/min with gradient generation as follow: 10–25% B (0–25 min), 25–35% B (25–35 min), 35–40% B (35–45 min), 40–55% B (45–75 min), 55–80% B (75–79 min), 80–90% B (79–82 min), 90–100% B (82–84 min), 100–10% B (84–87 min), and isocratic 10% B (87–90 min). Nitrogen gas nebulization was fixed at 45 psi at 5 L/min and 300 °C while the sheath gas was set at 11 L/min and 250 °C. The voltages for capillary and nozzle were fixed at 3.5 kV and 500 V, respectively. Mass scan within the range of 50–1300 *m*/*z* was utilized. MS/MS analyses were performed in automation with collision energy of 10, 15, and 30 eV for fragmentation purposes. Finally, peak identification was carried out in both positive and negative mode based on comparing fragmentation pattern with database. Instrument control data acquisition and processing were conducted using MassHunter Workstation software (Qualitative Analysis, version B.03.01) (Agilent Technologies).

### 2.9. Statistical Analysis

All the analyses and determinations were performed in triplicates and the results are presented as mean ± standard deviation (*n* = 3). The mean differences between different seaweed samples were analyzed by one-way analysis of variance (ANOVA) and Tukey’s honestly significant differences (HSD) multiple rank test at *p* ≤ 0.05. ANOVA was carried out via Minitab 19.0 software for windows. For correlations between polyphenol content and antioxidant activities, Pearson’s correlation coefficient at *p* ≤ 0.05, and multivariate statistical analysis including a principal component analysis (PCA), OriginPro 2024 was utilised.

## 3. Results and Discussion

### 3.1. Levels of Independent Extraction Variables for Free and Bound Phenolics Extraction

The levels for independent extraction variables for free and bound phenolic extraction were carried out according to a series of preliminary experiments (Tables S3 and S4). A significant increase of the seaweed free phenolics recovery was observed over the extraction amplitude of 20% to 80%, with the phenolics recovery reaching the maximum at an 80% amplitude. Beyond that amplitude range, the phenolic compound recovery and DPPH activity showed a slight decline. A similar trend was observed with an increase in the bound phenolic recovery and antioxidant activity. Based on these observations, 40%, 60%, and 80% amplitudes were chosen as the three design levels for free and bound phenolic extraction.

When the extraction time varied from 2 to 8 min, a remarkable increase in the TPC, TPhC, and DPPH activity was observed. Beyond that time range, there was a slight decrease in these properties. Therefore, 4, 6, and 8 min were chosen for the coded extraction time variable levels for free phenolics extraction. Similar preliminary experiments were conducted for bound phenolics. When the extraction time varied from 2 to 10 min, a consistent uptrend of the TPC and TPhC recovery and DPPH activity was noted. Therefore, 6, 8, and 10 min were chosen for the coded extraction time variable levels for bound phenolic extraction.

Free phenolics recovery significantly increased with the ratio of the solvent:seaweed sample, increasing from 10:1 to 20:1, and with a downward trend observed from 25:1 to 30:1. This trend is in line with the mass transfer kinetics whereby when a higher solvent:seaweed ratio is used, a steeper concentration gradient between the solid and liquid bulk is generated, producing a greater mass transfer driving force [12]. As a downward trend was observed with solvent:seaweed ratios of 25:1 to 30:1, the ratios of 10:1, 15:1, and 20:1 (mL:g) were selected as the three variable levels for the optimization process of free phenolic extraction.

The recovery of phenolics increased significantly when the NaOH concentration increased from 0.1 to 1.5 M with a downward trend observed from 1.5 to 2.0 M. The decrease in phenolic recovery at the higher NaOH concentration may be due to the high pH-induced degradation of phenolics [20]. Hence, the NaOH concentration at 0.5, 1.0, and 1.5 M were selected as the three variable levels for the optimization process of bound phenolic extraction.

### 3.2. Effect of Experimental Model on Free and Bound Phenolics Extraction

**Figure 1:**
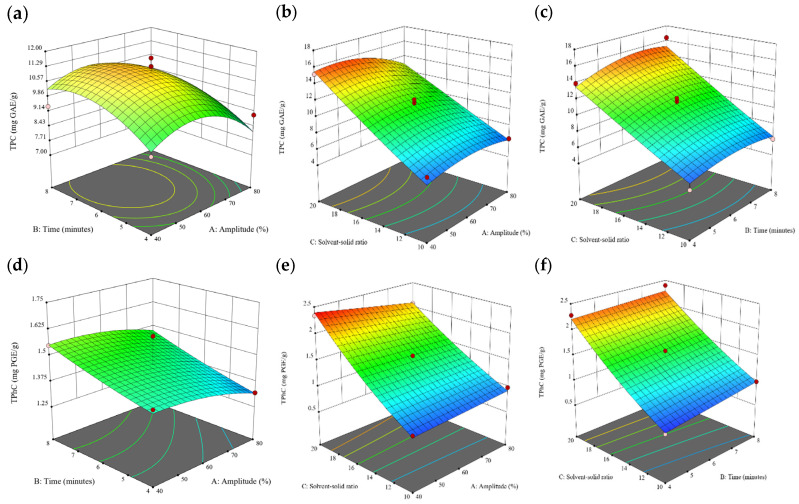

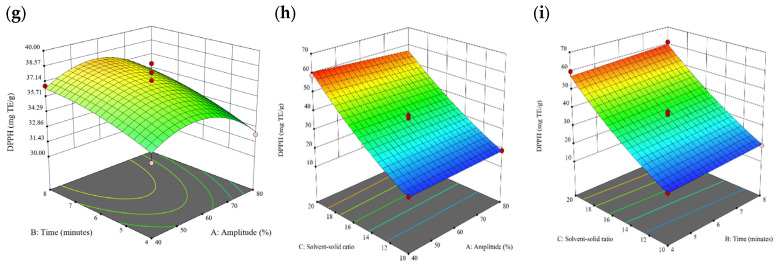
Three-dimensional response surface plots for total polyphenol content (**a**–**c**), total phlorotannin content (**d**–**f**), and 2,2-diphenyl-1-picrylhydrazyl (DPPH) assay (**g**–**i**) of free phenolic extract in *Cytospora* sp.

**Figure 2:**
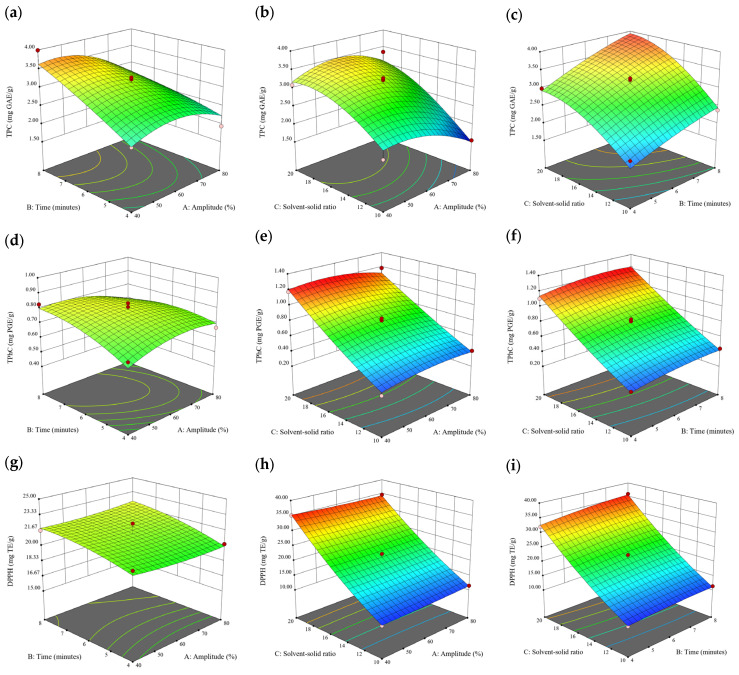
Three-dimensional response surface plots for total polyphenol content (**a**–**c**), total phlorotannin content (**d**–**f**), and 2,2-diphenyl-1-picrylhydrazyl (DPPH) assay (**g**–**i**) of free phenolic extract in *D. potatorum*.

**Figure 3:**
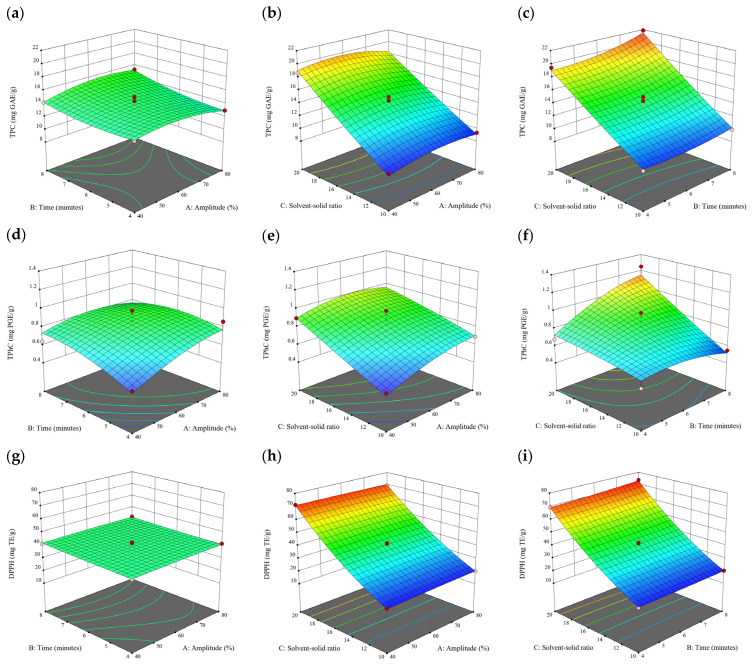
Three-dimensional response surface plots for total polyphenol content (**a**–**c**), total phlorotannin content (**d**–**f**), and 2,2-diphenyl-1-picrylhydrazyl (DPPH) assay (**g**–**i**) of free phenolic extract in *S. fallax*.

**Figure 4:**
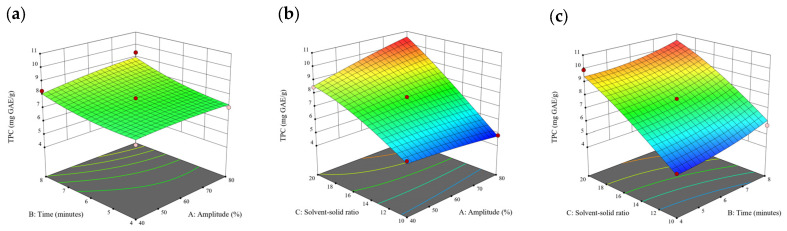

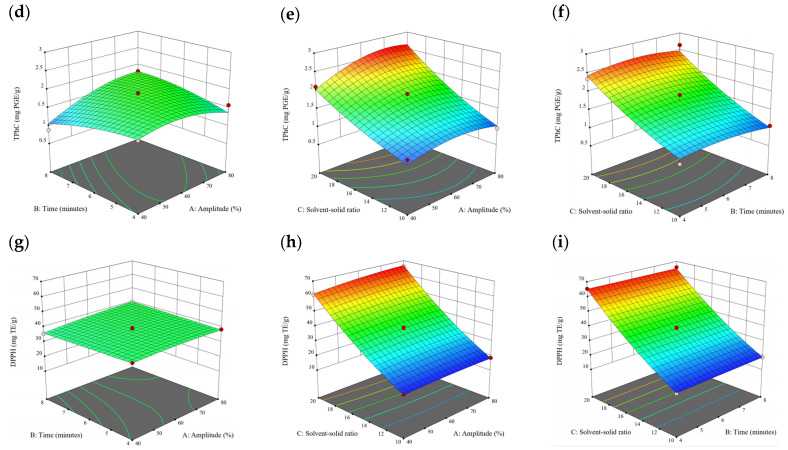
Three-dimensional response surface plots for total polyphenol content (**a**–**c**), total phlorotannin content (**d**–**f**), and 2,2-diphenyl-1-picrylhydrazyl (DPPH) assay (**g**–**i**) of free phenolic extract in *E. radiata*.

**Figure 5:**
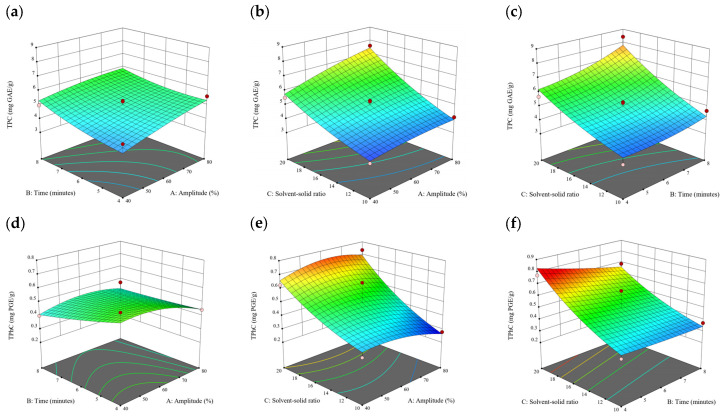

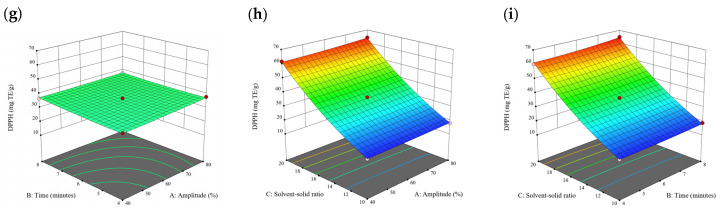
Three-dimensional response surface plots for total polyphenol content (**a**–**c**), total phlorotannin content (**d**–**f**), and 2,2-diphenyl-1-picrylhydrazyl (DPPH) assay (**g**–**i**) of free phenolic extract in *P. comosa*.

Based on the experimental results in Section 3.1, an experimental model based on BBD was implemented. Figure 1, Figure 2, Figure 3, Figure 4 and Figure 5 shows the results of the extraction of free phenolics from the five brown seaweed species according to the BBD. The different UAE conditions had a significant effect (*p* < 0.05) on the TPC, TPhC, and DPPH activity for the free phenolic extract of all five seaweed species. The analysis of the linear coefficients (A: amplitude, B: time, and C: solvent:solid ratio), quadratic coefficient (A^2^, B^2^, and C^2^), and the interaction coefficient (AB, AC, and BC) for the free phenolic extraction were recorded in Table 2. The linear effect of factor C was observed in the model developed for the TPC, TPhC, and DPPH activity for all free phenolic extracts of brown seaweeds, whereas factor B was only significant for the DPPH model for the free phenolic extract of *Cytospora* sp., the TPC and DPPH model for the free phenolic extract of *D. potatorum,* and the TPC model for the free phenolic extract of *S. fallax* and *E. radiata*. The linear effect of factor A was not significant for all the models developed except for an interactive effect between factor A and C observed in the free phenolic extract of *E. radiata*. Lastly, the quadratic coefficient (C^2^) was significant for the DPPH model for all free phenolic extracts of brown seaweeds as well as the TPhC model for the free phenolic extract of *Cytospora* sp.

### 3.3. Effect of Operational Parameters on the Extraction of Free and Bound Phenolics

A general uptrend of TPC, TPhC, and DPPH activity was observed when the ultrasonication amplitude increased from 40% to 80% during free phenolic extraction. A similar trend was noted during the bound phenolic extraction of *Cytospora* sp., *D. potatorum,* and *S. fallax*. During ultrasonication, high shear forces are generated which can disrupt the seaweed cell wall, promoting solvent penetration. The marginally lower TPC at a low ultrasonication amplitude may also be due to the formation of non-suitable bubbles which hinders the efficient mass transfer process [21]. Thus, a higher ultrasonication amplitude would result in the formation of effective cavitation bubbles which promotes the increased release of free and bound phenolics [21]. As the amplitude increases, the level of vibration intensity increases which leads to more cell wall disruption, allowing more solvent to permeate the cell wall to liberate free and bound phenolics [21]. However, a parabolic trend is observed in the TPC for *D. potatorum* and *S. fallax* free phenolic extract, in the TPhC for *E. radiata* and *P. comosa* free phenolic extract, as well as in the TPC, TPhC, and DPPH activity for *P. comosa* bound phenolic extract. In these models, the TPC, TPhC, or DPPH activity peaks around a 60% amplitude with a gradual decrease as the amplitude increases to 80%. This could be due to the formation of free radicals in the extraction solvent at a high amplitude which might lead to the degradation of free phenolics due to overheating of the solvent, in line with previous reports in studies with brown seaweed [22], pomegranate [23], and grape peel [24]. For bound phenolics, the disparity in the results might be due to differences in the cell wall composition between the brown seaweeds. It is postulated that at a 60% amplitude, the cell wall in *P. comosa* may be disrupted and phenolics were almost completely released. A further increase in amplitude would lead to the degradation of the phenolics due to high temperature and pressure.

With the increasing ultrasonication time, a notable rise in the TPC, TPhC, and DPPH activity was observed in the free phenolic extracts for all brown seaweed samples and bound phenolic extracts in *Cytospora* sp., *D. potatorum,* and *S. fallax*. This observed increase is indicative of the positive effect of the ultrasonication time on the recovery of free and bound phenolics from seaweed, which has been reported before [25,26]. Nonetheless, the TPC, TPhC, and DPPH activity increased from 6 to 8 min ultrasonication time, but decreased when 10 min of ultrasonication was applied in *E. radiata* and *P. comosa* bound phenolic extraction. This discrepancy can be attributed to the different cell wall composition and structure that could be present in these two seaweed samples which may require a shorter ultrasonication time for the near-complete release of phenolic compounds. Thus, an extended period of ultrasonication would lead to the degradation of phenolics.

The TPC, TPhC, and DPPH activity in the free phenolic extract of all brown seaweed samples show an increasing trend when the solvent:solid ratio increases from 10:1 to 20:1, as a higher amount of solvent enhances the cell wall penetration by increasing the extent of swelling in the cell wall and membrane [25]. This results in a stronger interaction between the solvent and free phenolic compounds that are polar in nature, thus causing the greater solubilization of the free phenolics into the solvent [21]. According to Fick’s law, the higher concentration gradient between the cell wall content and extraction medium drives the yield of bioactive compounds [27]. Indeed, based on the ANOVA analysis in Table 2, the effect of the solvent:seaweed ratio is statistically significant (*p* < 0.05) compared to the other factors supporting it as a major variable during the free phenolic extraction process. With the increasing NaOH concentration, a general uptrend of the TPC, TPhC, and DPPH activity was observed in the bound phenolic extract of all brown seaweed samples except for *E. radiata*. Increasing the NaOH concentration helps to enhance its ability to break bonds between phenolic compounds and the cell wall matrix, thus achieving a higher extraction efficiency [28,29].

### 3.4. Optimization and Verification of Extraction Conditions for Free and Bound Phenolics

The optimum extraction conditions were determined and used for calculating the predicted values of response variables using the prediction equations derived by the response surface methodology. Verification experiments were conducted at the predicted conditions to demonstrate that the experimental values were within the confidence range of the predicted values, thus confirming the validity and adequacy of the predicted models. The optimum conditions and results from the verification experiments for each of the seaweed samples are shown in Table 4. Based on the results shown in Table 4, the experimental values were within a 95% confidence range of the predicted results, thus verifying the models used to optimize the extraction of free and bound phenolics from brown seaweed. The optimized extraction condition differs between different brown seaweed species due to variation in the cell wall composition and structure among the brown seaweeds [14,30]. Thus, the extraction condition will vary depending on the cell wall composition and structure of brown seaweeds.

### 3.5. Comparison between Conventional and Ultrasonic Extraction of Phenolic Compounds

The free and bound phenolics in brown seaweed obtained by conventional and ultrasonic extraction are shown in Table 5 The wide disparity in the TPC, TFC, and TCT among all five seaweed species may arise due to multiple abiotic and biotic factors such as the species, plant developmental stage, size, depth, salinity, light exposure, etc., [31]. UAE had a significant effect (*p* < 0.05) in increasing the extraction of free phenolics by 1–2-folds from the brown seaweeds (Table 5). Compared to the conventional extraction method, UAE produce the highest increase in TPC (two-fold) from *P. comosa,* while the lowest increase (1.1-fold) was from *D. potatorum*. These results corroborate the findings of previous report that showed significantly higher extraction of free phenolics when UAE treatment was applied on seaweed (Ummat, Tiwari, Jaiswal, Condon, Garcia-Vaquero, O’Doherty, O’Donnell, and Rajauria [18]; Dang et al. [32]). As shown in Table 5, the TFC, TCT, and TPhC obtained by UAE were higher compared to conventional extraction for the free phenolics. In particular, the effect of UAE is more effective in extracting free tannins from brown seaweed. Under the conventional extraction method, free tannins were not detected in the free phenolic extracts of *D. potatorum*, *S. fallax,* and *P. comosa*.

The highest amount of extraction of free phenolics (TPC) was obtained for *Sargassum fallax* (17.43 ± 0.02 mg GAE/g, conventional; 20.32 ± 0.41 mg GAE/g, ultrasonication) and *Cytospora* sp. (10.62 ± 0.24 mg GAE/g, conventional; 14.64 ± 1.12 mg GAE/g, ultrasonication). However, the highest amount of extraction of free phlorotannins (TPhC) was obtained for *Cytospora* sp. (1.22 ± 0.04 mg PGE/g, conventional; 2.42 ± 0.23 mg PGE/g, ultrasonication) and *E. radiata* (2.03 ± 0.03 mg PGE/g, conventional; 2.64 ± 0.15 mg PGE/g, ultrasonication). The disparity between the TPC and TPhC values could be due to the Folin–Ciocalteu method in the determination of the TPC, as the reagent can be reduced by other reducing compounds other than phenolics in the extracts, such as ascorbic acids, monosaccharides, and proteins [33]. Hence, the overestimation of the phenolic content using the Folin–Ciocalteu method can occur with crude extracts. The DMBA reagent used for the determination of phlorotannin is more specific in its chemistry, deriving from the reaction between DMBA and phloroglucinol units present in phlorotannin.

In brown algae, phlorotannin is postulated to be part of the cell wall system whereby it forms complexes with alginate that contribute to the cell wall defence system [34]. Thus, extraction using an organic solvent is not strong enough in disrupting the phlorotannin–alginate complex in releasing phlorotannin from the seaweed cell wall. Alkaline hydrolysis breaks down the wall materials and helps to release and solubilize bound phenolic compounds [16]. The phenolic content of bound phenolics in all the seaweed species in this study were found to be lower than that of the free phenolics, which was similarly reported before for seaweeds [8,35]. However, Wu, Gao, Wang, Peng, Guo, Ma, Zhang, Zhang, Wu, and Xiao [16] reported a higher content of bound phenolics compared to free phenolics in *Sargassum* sp. The disparity could be due to differences in the sample collections, for example, samples collected in dry seasons are exposed to higher solar radiation and the seaweed may have accumulated more cell-wall-bound phlorotannin for protection against UV damage [16]. As expected, UAE treatment increases the extraction yield of bound phenolics from our brown seaweed samples compared to conventional extraction (*p* < 0.05), which is consistent with the current literature [28,29].

To comprehensively evaluate the antioxidant property of the extracted phenolics from the brown seaweed, radical scavenging (DPPH and ABTS), reducing (FRAP and PRAC), and metal chelating (FICA) levels were determined (Table 5). Overall, the antioxidant capacity of the seaweed extract using UAE was significantly higher than with conventional extraction (*p* < 0.05). The observed trend for the DPPH and ABTS activity correlated with the TPC for the brown seaweed species; the highest DPPH activity (49.97 ± 1.14 mg TE/g, conventional; 73.66 ± 0.51 mg TE/g) and ABTS activity (50.40 ± 1.80 mg TE/g, conventional; 86.88 ± 0.40 mg TE/g) were observed for *S. fallax*. Similarly, *S. fallax* had the highest FRAP and PRAC. The positive correlation between the TPC and DPPH and ABTS activity of the seaweed extracts confirms what was previously reported [36,37].

The trend observed in FICA differs from the DPPH and ABTS, with extracted phenolics from *Cytospora* sp. exhibiting a higher metal chelating activity than *S. fallax*. The FICA is based on the ability of the phenolic compound to chelate ferrous ion, thus preventing it from participating in the pro-oxidant Fenton reactions which generate free radicals such as reactive hydroxyl radicals [38]. The metal chelating ability of phenolic compounds is structure-dependent, and the present of the catechol motif enables metal chelation by the adjacent di-hydroxy groups [38]. Based on the current results, it can be concluded that the phenolic profile in *S. fallax* has a greater hydrogen donating ability and reducing power whereas the phenolic profile in *Cytospora* sp. has a greater metal chelating ability.

For the bound phenolic extract, the highest DPPH and ABTS activity were detected in *D. potatorum* and *Cytospora* sp. (Table 5). For ultrasonic extraction, a consistent trend whereby the highest DPPH activity, ABTS activity, FRAP, PRAC, and FICA were detected in *Cytospora* sp. However, in conventional extraction, *Cytospora* sp. only exhibited the highest activity in FRAP, PRAC, and FICA, whereas *D. potatorum* had the highest DPPH and ABTS activity. The disparity observed in the trend between conventional and ultrasonic extraction may arise from the higher extraction efficiency of ultrasonic treatment that helps extract more phenolic compounds in *Cytospora* sp. which confers to their subsequent higher antioxidant activity. This is in line with the study by Sun, Zhao, Wang, Tan, Shi, Sedjoah, Shao, Li, Wang, and Wan [26] and Zhong, Zhang, Wang, Yang, Li, Zhu, and Liu [29] that showed overall higher antioxidant activity of seaweed extracts recovered from UAE.

The extraction of phenolic compounds using UAE has a clear advantage over conventional extraction due to the higher phenolic content and antioxidant activities. In addition, the established UAE shortens the extraction time and energy consumption which translates to lower operational costs. This UAE technology can be further applied to various industrial applications such as the cosmetic or nutraceutical field to increase extraction efficiency in a shorter amount of time and at a lower cost.

### 3.6. Correlation among Phenolic Content and Antioxidant Activity

**Figure 11:**
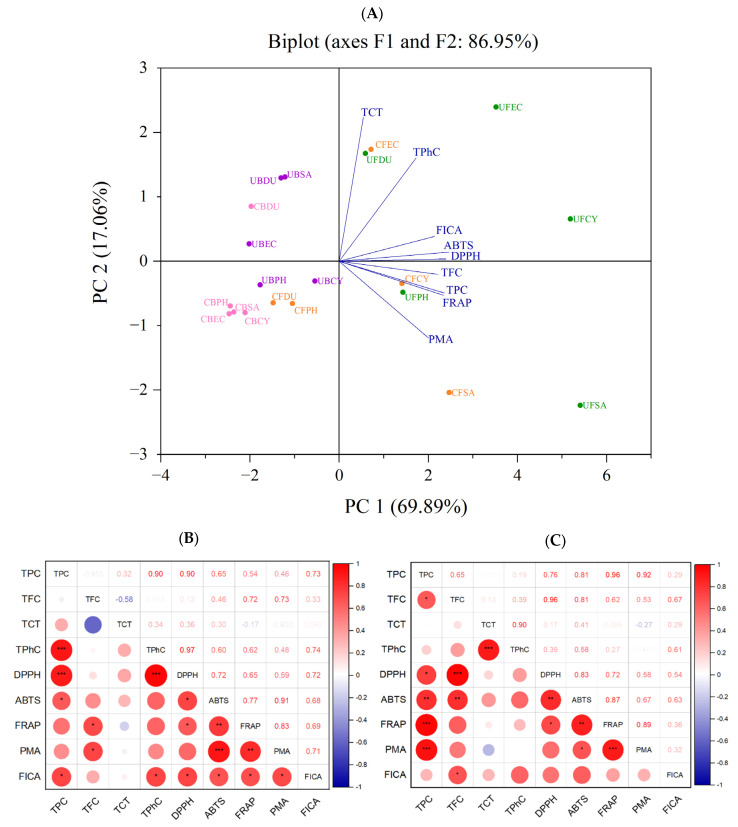
Correlation analysis of antioxidant activity. (**A**) PCA analysis. (**B**) Correlation plot of antioxidant activities of free phenolics. (**C**) Correlation plot of antioxidant activities of bound phenolic extract (* *p* ≤ 0.05, ** *p* ≤ 0.01, *** *p* ≤ 0.001). TFC (total flavonoid content), TPC (total phenolic content), TCT (total condensed tannin), TPhC (total phlorotannin content), ABTS (2,2′-azino-bis(3-ethylbenzothiazoline-6-sulfonic acid), DPPH (2,2-diphenyl-1-picrylhydrazyl), FRAP (ferric-reducing antioxidant power), PRAC (phosphomolybdate-reducing antioxidant capacity), FICA (ferrous ion chelating activity), CF (Conventional Free), UF (Ultrasonication Free), CB (Conventional Bound), UB (Ultrasonication-Bound), CY (*Cytospora* sp.), DU (*Durvillaea potatorum*), SA (*Sargassum fallax*), EC (*Ecklonia radiata*), PH (*Phyllospora comosa*).

According to the analysis and results shown above, different extraction methods (conventional and ultrasonication) gave rise to a significant effect on the phenolic content and antioxidant capacity of the seaweed extracts. Principal component analysis (PCA) was used to reduce the size of large sets of variables into smaller sets (principal components) to explain the disparity within the original set of variables [39]. Based on the loaded values and factor map, the FICA, ABTS, DPPH, TFC, TPC, and FRAP are the main contributors to PC1, whereas PC2 is mainly attributed to the TCT, DMBA, and PRAC. In the PCA scoring chart (Figure 11), the extracted principal components PC1 and PC2 were 69.89% and 17.06%, respectively. All the phenolic contents and antioxidant capacities were in the positive direction of the *x*-axis. Specifically, the TPC and FRAP were highly correlated, as expected, since phenolic compounds contributed to the reducing property. According to the PCA plot, the positionings of the free phenolic extracts of brown seaweeds, obtained using both conventional and ultrasonic extraction, are widely dispersed. The conventionally extracted bound phenolics of all brown seaweeds (except *D. potatorum*) were tightly clustered together, indicating a similar phenolic content and antioxidant activity. However, when ultrasonication was applied for bound phenolic extraction, the positioning of the samples was more dispersed.

A more detailed overview of the correlation between the antioxidant activity assays is illustrated in the correlation plot in Figure 11. The TPC of free phenolics are positively correlated with DPPH, ABTS, FRAP, PMA, and FICA, with the weakest correlation observed between TPC and FICA (*r* = 0.29). A similar positive correlation was observed between the TPC of bound phenolics and the DPPH, ABTS, FRAP, PMA, and FICA, with a stronger FICA correlation at r = 0.73. The disparity in the correlation strength of TPC and FICA between the free and bound extract may be attributed to the varying phenolic profiles in these extracts. The results from this study show that the antioxidant activity of the seaweed extracts is mainly attributed to the TPC, which is in line with the results reported by many researchers [16,40,41]. These results also provide further support showing the important role of seaweed polyphenol as a strong radical scavenger.

### 3.7. Heatmap Analysis of Phenolic Compounds

**Figure 12:**
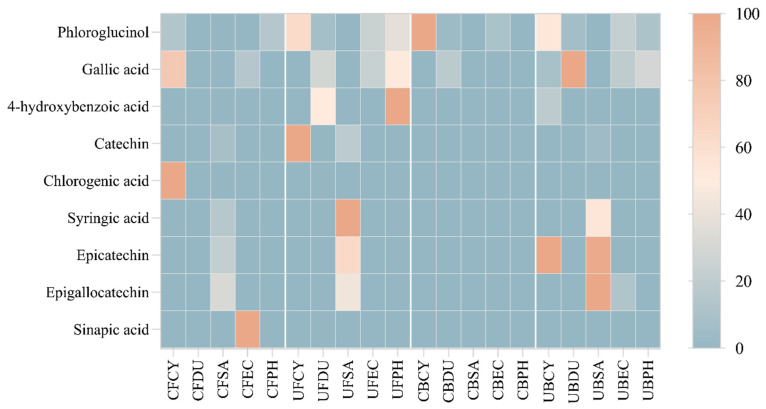
Heat map distribution of free and bound phenolic extract of brown seaweed samples using conventional and ultrasonication extraction methods. CF, Conventional Free; UF, Ultrasonication Free; CB, Conventional Bound; UB, Ultrasonication-Bound; CY, *Cytospora* sp.; DU, *Durvillaea potatorum*; SA, *Sargassum fallax*; EC, *Ecklonia radiata*; PH, *Phyllospora comosa*. Results are expressed in mg/mL.

The regression equation, correlation coefficient, and fitness of calibration model were analyzed and are shown in Table S5. The HPLC-PDA data were analyzed and constructed into a hierarchical heat map showing the distribution of phenolic compounds across the free and bound phenolic extracts of the brown seaweeds (Figure 12). The varying color in the heatmap shows the concentration of phenolic compounds. In particular, phloroglucinol, gallic acid, and 4-hydroxybenzoic acid are detected in most brown seaweed samples (free and bound extracts). Gallic acid is produced in brown seaweed via the dehydrogenation of 5-dehydroshikimic acid [15] and this compound was detected in the free phenolic extract of *Cytospora* sp., *D. potatorum*, *E. radiata*, and *P. comosa,* as well as the bound phenolic extract of *D. potatorum*, *E. radiata,* and *P. comosa*. Chlorogenic acid and sinapic acid were only detected in the conventional free phenolic extract of *Cytospora* sp. and *P. comosa,* respectively. The absence of these compounds in the ultrasonic phenolic extract may be due to the thermal sensitivity nature of these compounds, leading them to potentially be degraded under ultrasonic conditions. As can be seen from the heatmap, a higher concentration of phenolic compounds was detected in the free and bound phenolic extract obtained by ultrasonication, which corroborates with the results from antioxidant assays.

### 3.8. LC-ESI-QTOF-MS/MS Characterization

Table 6 shows the phenolic compounds detected in the free and bound phenolics from brown seaweeds by LC-ESI-QTOF-MS/MS. From the MS and MS/MS spectra, a total of 25 free and bound phenolic compounds, which include phenolic acids, flavonoids, lignans, and other polyphenols, were identified based on their retention times, molecular weights, and *m*/*z* value of molecular ions.

Hydroxycinnamic acids were detected in the free phenolic extracts from *Cytospora* sp., *D. potatorum*, *E. radiata* and *P. comosa* and bound phenolic extracts from *Cytospora* sp. and *E. radiata*. Based on the MS/MS analysis, Compound **1** (1,2,2′-triferuloylgentiobiose) and Compound **3** (1-sinapoyl-2,2′-diferuloylgentiobiose) were detected in *E. radiata*. Compound **1** was confirmed by the presence of product ions at *m*/*z* 693 and *m*/*z* 517 due to loses of pentose moiety and CO_2_ from the parent ion. Compound **1** was detected in both the conventional and ultrasonic extraction of free phenolics from *E. radiata* but Compound **3** was only detected from the UAE of bound phenolics in *E. radiata*. The results of the LC-MS/MS analysis corroborated with the previous findings of hydroxycinnamic in other brown seaweed species (*Sargassum wightii*, *Ulva rigida*, and *Gracilaria edulis*) [42]. The presence of Compound **2** (p-coumaroyl malic acid) was detected in *Cytospora* sp., *D. potatorum,* and *P. comosa*, which were also reported by Subbiah, Ebrahimi, Agar, Dunshea, Barrow, and Suleria [15].

Hydroxybenzoic acid derivatives in brown seaweed samples were identified as 3,4-*O*-dimethylgallic acid (*m*/*z* 199.061) and 4-*O*-methylgallic acid (*m*/*z* 185.0454). The presence of 4-*O*-methylgallic acid was confirmed by the product ions at *m*/*z* 170 and *m*/*z* 142, indicating the loss of CH_3_ and CO_2_ from the precursor ions, respectively. 3,4-*O*-Dimethylgallic acid was detected in both the free and bound extract of all brown seaweed species except for *E. radiata*. The presence of hydroxybenzoic acid in seaweed has also been reported previously. For example, Agregán et al. [43] identified a hydroxybenzoic acid derivative present in *Ascophyllum nodosum* brown seaweed free phenolic extract. Rajauria et al. [44] detected *m*-hydroxybenzaldehyde, *p*-hydroxybenzaldehyde, gallic acid, and gallic acid 4-*O*-glucoside by MS/MS analysis, which corresponds to the hydroxybenzoic acid derivatives in the brown seaweed (*Himanthalia elongate*) free phenolic extract.

The presence of flavonoids in seaweeds is intriguing as it is believed that flavonoids evolved from terrestrial to aquatic plants to mitigate the increased UV exposure for the marine environment, as stated by Subbiah, Ebrahimi, Agar, Dunshea, Barrow, and Suleria [15]. In algae, most flavonoids are naturally found as the glycoside derivatives of the aglycones similar to those in plants [45]. The flavonoids detected in the brown seaweeds in our study included flavonol, anthocyanin, flavonone, flavone, and isoflavonoids, and these groups of flavonoids are also present in plants. Among them, peonidin 3-*O*-diglucoside-5-*O*-glucoside and hesperidin 5,7-*O*-diglucuronide are unique compounds that were identified as the bound phenolic extract of *P*. comosa and the free phenolic extract of *E*. radiata and *D. potatorum,* which are not reported in plants.

Compound **6**, quercetin 3-*O*-xylosyl-glucuronide (*m*/*z* 611.1223), has product ions at *m*/*z* 479, 303, 285, and 239 in the MS/MS spectrum that can be attributed to the loss of pentose, glucuronide, and water from the precursor ion. It is a glycosylated flavonol, whereby the quercetin is substituted with a xylose-glucuronide disaccharide on the C3 position of the aglycone. This compound was found in both the conventional and ultrasonic extraction of free phenolic from *D. potatorum* and *S. fallax*.

Compound **14**, hesperetin 5,7-*O*-diglucuronide, was detected in the free phenolic extract of *E. radiata* and *D. potatorum*. This compound was previously detected as hesperidin metabolites in the plasma and urine of human subjects after the consumption of orange juice and fruits [46], and has demonstrated anti-inflammation, anticancer, anti-allergic, would healing, and neuroprotective properties [47]. Its presence in brown seaweeds presents an interesting source for this bioactive entity [48].

Flavonoid glucosides are glycosylated flavonoid compounds commonly found in plant [17]. We detected spinacetin 3-*O*-glucosyl-(1->6)-glucoside and peonidin 3-*O*-diglucoside-5-*O*-glucoside in the free phenolic extract of *D. potatorum* and the bound phenolic extract of *P. comosa*. The presence of flavonoid glycoside in seaweed is not new and has been reported by previous researchers. For example, quercetin 3-*O*-neophesperidosid was identified in green algae (*Tetraselmis suecica* and *Nannochloropsis gaditana*) [49].

Three anthocyanins were identified in the brown seaweed, which included cyanidin 3-*O*-(6″-p-coumaroyl-glucoside), cyanidin 3-*O*-diglucoside-5-*O*-glucoside, and peonidin 3-*O*-diglucoside-5-*O*-glucoside. Cyanidin-3-*O*-glucoside, which is similar, has been identified in *Himanthalia elongata* [50]. Other phenolic compounds belonging to the classes of hydroxycoumarin, hydroxyphenylpropene, hydroxybenzaldehyde, phenolic terepene, and curcuminoid were also detected in the seaweed samples.

## 4. Conclusions

The development of an appropriate extraction method for the efficient recovery of phenolic compounds from brown seaweed is essential for the food and nutraceutical industries. In this study, the optimization of UAE extraction parameters (amplitude, time, solvent:solid ratio, and NaOH concentration) from brown seaweeds was conducted using BBD. The results from this study showed that UAE is an effective extraction technique for brown seaweed phenolic compounds as compared to the conventional extraction method. Ultrasonication led to a reduced extraction time, higher phenolic contents, and a higher level of antioxidant activity. Based on the correlation analysis conducted, it is also noted that the TPC in the phenolic extract is highly correlated to the resulting antioxidant activities, such as the DPPH radical scavenging activity and FRAP reducing power. The characterization and profiling of phenolic compounds in a brown seaweed extract using LC-ESI-QTOF-MS/MS facilitated a deeper and more comprehensive overview of the types of phenolic compounds present in brown seaweed. A natural progression to this work is to identify further biological activities of seaweed phenolics, and this could pave a way for the utilization of brown seaweed in the food and nutraceutical industries.

## Figures and Tables

**Figure 6:**
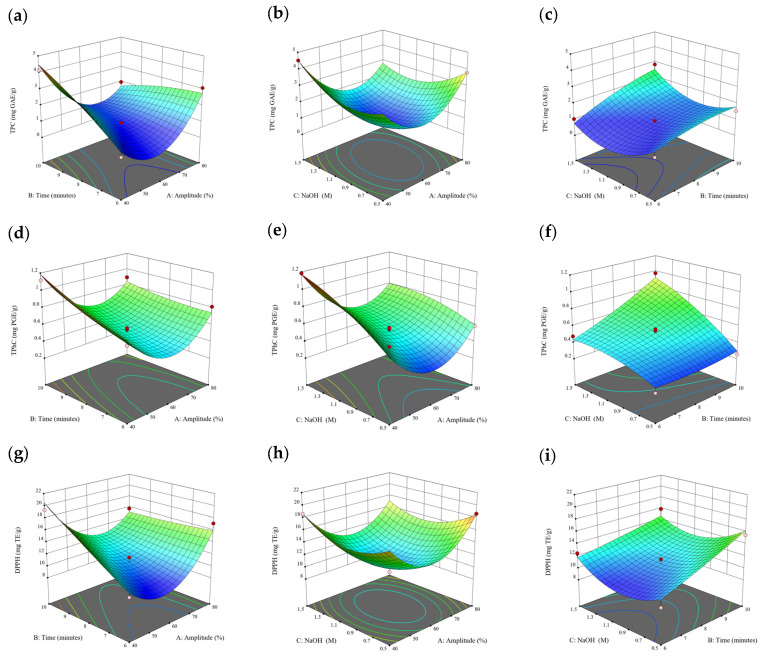
Three-dimensional response surface plots for total polyphenol content (**a**–**c**), total phlorotannin content (**d**–**f**), and 2,2-diphenyl-1-picrylhydrazyl (DPPH) assay (**g**–**i**) of bound phenolic extract in *Cytospora* sp.

**Figure 7:**
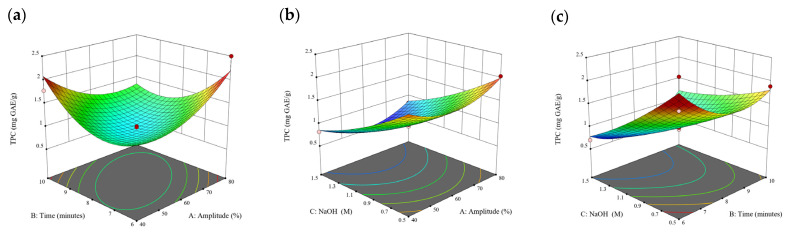

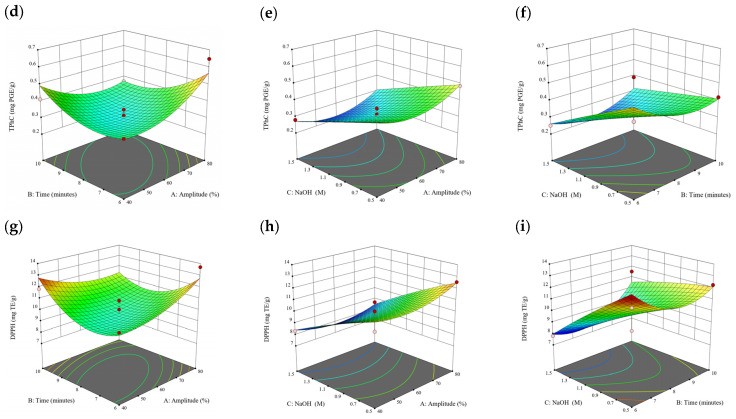
Three-dimensional response surface plots for total polyphenol content (**a**–**c**), total phlorotannin content (**d**–**f**), and 2,2-diphenyl-1-picrylhydrazyl (DPPH) assay (**g**–**i**) of bound phenolic extract in *D. potatorum*.

**Figure 8:**
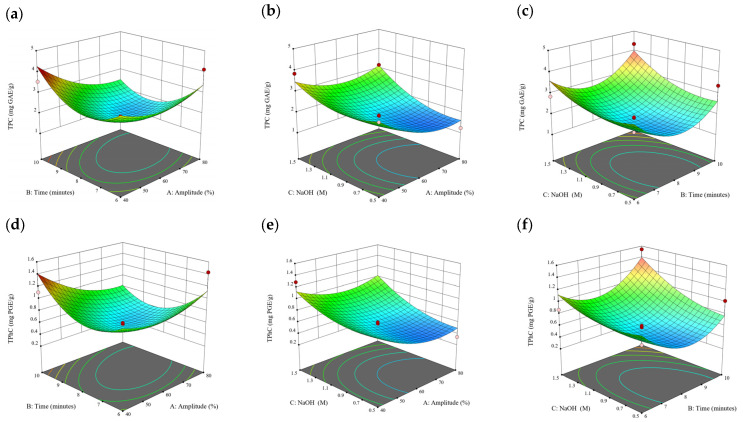

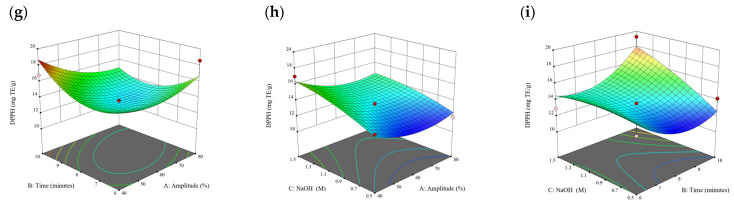
Three-dimensional response surface plots for total polyphenol content (**a**–**c**), total phlorotannin content (**d**–**f**), and 2,2-diphenyl-1-picrylhydrazyl (DPPH) assay (**g**–**i**) of bound phenolic extract in *S. fallax*.

**Figure 9:**
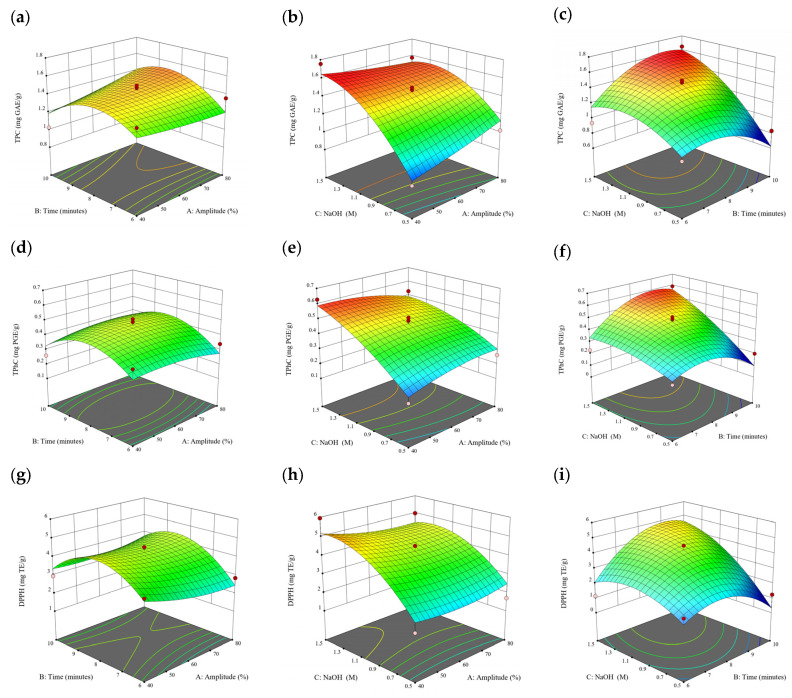
Three-dimensional response surface plots for total polyphenol content (**a**–**c**), total phlorotannin content (**d**–**f**), and 2,2-diphenyl-1-picrylhydrazyl (DPPH) assay (**g**–**i**) of bound phenolic extract in *E. radiata*.

**Figure 10:**
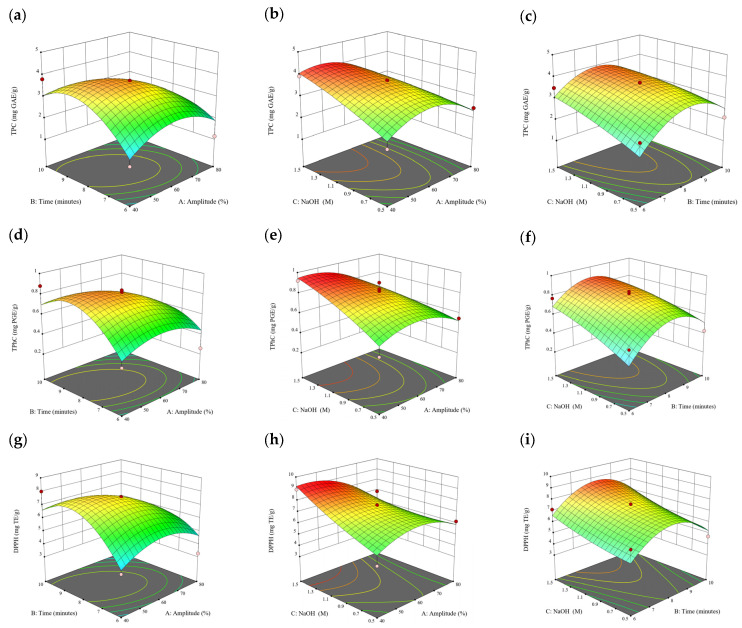
Three-dimensional response surface plots for total polyphenol content (**a**–**c**), total phlorotannin content (**d**–**f**), and 2,2-diphenyl-1-picrylhydrazyl (DPPH) assay (**g**–**i**) of bound phenolic extract in *P. comosa*.

**Table 1:** Independent variables and response values for free and bound phenolic extractions.

Symbols	Independent Variables	−1	0	1
Free phenolic extraction				
X_1_	Amplitude (%)	40	60	80
X_2_	Time (min)	4	6	8
X_3_	Solvent:solid ratio	10	15	20
Y_1_	TPC (mg GAE/g)			
Y_2_	TPhC (mg PGE/g)			
Y_3_	DPPH (mg TE/g)			
Bound phenolic extraction				
X_1_	Amplitude (%)	40	60	80
X_2_	Time (min)	6	8	10
X_3_	NaOH concentration (M)	0.5	1.0	1.5
Y_1_	TPC (mg GAE/g)			
Y_2_	TPhC (mg PGE/g)			
Y_3_	DPPH (mg TE/g)			

Abbreviations: GAE, gallic acid equivalents; PGE, phloroglucinol equivalents; TE, Trolox equivalents; TPC, total phenolic content; TPhC, total phlorotannin content; DPPH, 2,2-diphenyl-1-picrylhydrazyl radical scavenging activity.

**Table 2:** Analysis of variance for the independent variables for free phenolics by the experimental treatments.

*p*-Value	*Cytospora* sp.			*D. potatorum*			*S. fallax*			*E. radiata*			*P. comosa*		
	TPC	TPhC	DPPH	TPC	TPhC	DPPH	TPC	TPhC	DPPH	TPC	TPhC	DPPH	TPC	TPhC	DPPH
Model	0.01	0.00	<0.0001	0.04	0.00	<0.0001	0.00	0.14	<0.0001	0.00	0.01	<0.0001	0.01	0.02	<0.0001
A	0.31	0.27	0.27	0.11	0.72	0.95	0.90	0.17	0.93	0.14	0.11	0.01	0.12	0.59	0.08
B	0.30	0.08	0.07	0.02	0.09	0.02	0.04	0.20	0.11	0.01	0.89	0.84	0.12	0.25	0.07
C	0.00	<0.0001	<0.0001	0.00	<0.0001	<0.0001	<0.0001	0.01	<0.0001	<0.0001	0.00	<0.0001	0.00	0.00	<0.0001
A*B	0.93	0.91	0.86	0.25	0.17	0.65	0.32	0.40	0.82	0.37	0.08	0.06	0.60	0.51	0.39
A*C	0.30	0.11	0.26	0.34	0.50	0.38	0.44	0.50	0.34	0.03	0.33	0.04	0.43	0.46	0.58
B*C	0.55	0.58	0.93	0.79	0.88	0.16	0.28	0.11	0.15	0.67	0.56	0.99	0.40	0.61	0.25
A^2^	0.15	0.62	0.06	0.15	0.16	0.64	0.08	0.38	0.65	0.67	0.10	0.13	0.72	0.41	0.59
B^2^	0.32	0.87	0.60	0.67	0.22	0.39	0.22	0.44	0.65	0.28	0.43	0.72	0.59	0.94	0.51
C^2^	0.84	0.05	0.03	0.14	0.40	0.02	0.59	0.93	0.00	0.25	0.40	0.00	0.50	0.48	0.00
Lack of fit	0.28	0.76	0.14	0.20	0.46	0.74	0.51	0.59	0.68	0.54	0.28	0.39	0.22	0.79	0.74
R^2^	0.95	0.99	1.00	0.91	0.99	1.00	0.99	0.83	1.00	0.98	0.95	1.00	0.89	0.87	1.00
Adjusted R^2^	0.93	0.97	0.99	0.75	0.96	0.99	0.96	0.53	1.00	0.94	0.87	1.00	0.68	0.63	1.00
Adeq. Precision	9.33	20.03	32.15	8.40	18.68	39.97	21.10	6.21	54.10	15.93	9.69	72.18	6.91	6.82	48.20

A—Amplitude (%); B—Time (min); C—solvent:solid ratio; R^2^: coefficient of determination; TPC, total phenolic content; TPhC, total phlorotannin content; DPPH, 2,2-diphenyl-1-picrylhydrazyl radical scavenging activity. Figure 6, Figure 7, Figure 8, Figure 9 and Figure 10 shows the results of response variables for bound phenolics extraction of all five brown seaweed species. Significant effect (*p* < 0.05) was observed for TPC, TPhC, and DPPH activity between different UAE conditions for all bound phenolic extracts of brown seaweed. The analysis of variance for linear coefficients (A: amplitude, B: time, and C: NaOH concentration), quadratic coefficient (A^2^, B^2^, and C^2^), and the interaction coefficient (AB, AC, and BC) for bound phenolic extraction are recorded in Table 3. The linear effect of B was significant in the model developed for TPC, TPhC, and DPPH activity for bound phenolic extract of *Cytospora* sp., whereas factor C significantly affected the TPC, TPhC, and DPPH activity for bound phenolic extract of *D. potatorum* and *E. radiata* and TPhC for bound phenolic extract of *Cytospora* sp. and *S. fallax*. Interactive effect between factors A*B and B*C as well as quadratic effect A^2^, B^2^, and C^2^ were also significant in the models developed, as can be seen in Table 3. High R^2^, Adj-R^2^, and the non-significant value of lack of fit confirmed that the TPC, TPhC, and DPPH for both free and bound phenolic extracts can be predicted according to the mathematical model of equation generated.

**Table 3:** Analysis of variance for the independent variables for bound phenolics by the experimental treatments.

*p*-Value	*Cytospora* sp.			*D. potatorum*			*S. fallax*			*E. radiata*			*P. comosa*		
	TPC	TPhC	DPPH	TPC	TPhC	DPPH	TPC	TPhC	DPPH	TPC	TPhC	DPPH	TPC	TPhC	DPPH
Model	0.01	0.01	0.05	0.01	0.01	0.03	0.07	0.11	0.15	0.03	0.04	0.02	0.05	0.11	0.07
A	0.20	0.06	0.87	0.77	0.76	0.61	0.10	0.23	0.28	0.56	0.91	0.62	0.07	0.11	0.09
B	0.03	0.02	0.03	0.77	0.96	0.72	0.98	0.78	0.58	0.63	0.48	0.29	0.60	0.63	0.62
C	0.75	0.00	0.92	0.00	0.00	0.00	0.07	0.05	0.06	0.00	0.00	0.00	0.12	0.08	0.06
A*B	0.01	0.21	0.05	0.03	0.07	0.14	0.20	0.19	0.18	0.39	0.62	0.41	0.11	0.23	0.15
A*C	0.09	0.15	0.51	0.86	0.87	0.50	0.44	0.62	0.76	0.29	0.34	0.62	0.23	0.34	0.19
B*C	0.32	0.09	0.50	0.13	0.12	0.12	0.57	0.39	0.13	0.05	0.07	0.05	0.49	0.57	0.86
A^2^	0.00	0.00	0.01	0.05	0.11	0.27	0.14	0.20	0.21	0.87	0.53	0.55	0.03	0.15	0.09
B^2^	0.51	0.62	0.90	0.01	0.02	0.04	0.01	0.02	0.05	0.05	0.02	0.01	0.01	0.02	0.01
C^2^	0.02	0.30	0.06	0.47	0.38	0.96	0.18	0.29	0.60	0.07	0.34	0.06	0.67	0.65	0.68
Lack of fit	0.27	0.22	0.27	0.10	0.32	0.26	0.27	0.15	0.09	0.41	0.38	0.57	0.42	0.25	0.36
R^2^	0.95	0.95	0.90	0.89	0.90	0.86	0.80	0.80	0.84	0.86	0.84	0.86	0.83	0.77	0.81
Adjusted R^2^	0.85	0.85	0.72	0.76	0.76	0.67	0.74	0.78	0.76	0.68	0.75	0.68	0.78	0.68	0.76
Adeq. Precision	8.55	10.25	6.33	7.28	7.98	7.24	5.30	4.84	4.97	7.88	7.44	8.07	6.43	5.54	6.97

A—amplitude (%); B—Time (min); C—NaOH concentration (M); R^2^: coefficient of determination; TPC, total phenolic content; TPhC, total phlorotannin content; DPPH, 2,2-diphenyl-1-picrylhydrazyl radical scavenging activity.

**Table 4:** Experimental and predicted values of response variables at optimum extraction conditions for free and bound phenolics.

Samples	Optimal Extraction Conditions	Values	TPC (mg GAE/g)	TPhC (mg PGE/g)	DPPH (mg TE/g)
Free Phenolics					
*Cytospora* sp.	Amplitude 52%	Predicted	15.57	2.34	60.64
	Time 8 min	Experimental	14.64 ± 1.12	2.42 ± 0.23	60.67 ± 0.17
	Solvent–solid ratio 20	95% CI	12.21–19.34	2.13–2.55	56.40–65.03
*Durvilleae potatorum*	Amplitude 51%	Predicted	3.92	2.48	35.91
	Time 8 min	Experimental	4.01 ± 0.29	0.43 ± 0.02	35.26 ± 0.08
	Solvent–solid ratio 20	95% CI	3.08–4.77	2.08–2.88	33.18–38.68
*Sargassum fallax*	Amplitude 57%	Predicted	21.46	1.20	73.07
	Time 8 min	Experimental	20.32 ± 0.41	1.28 ± 0.02	73.66 ± 0.51
	Solvent–solid ratio 20	95% CI	19.76–23.16	18.87–24.05	68.90–77.24
*Ecklonia radiata*	Amplitude 80%	Predicted	11.51	2.76	67.46
	Time 8 min	Experimental	11.6 ± 0.57	1.01 ± 0.02	66.84 ± 0.34
	Solvent–solid ratio 20	95% CI	10.16–12.87	2.13–3.39	64.19–70.73
*Phyllospora comosa*	Amplitude 80%	Predicted	8.33	0.83	65.69
	Time 8 min	Experimental	8.15 ± 0.20	0.94 ± 0.01	64.38 ±0.80
	Solvent–solid ratio 20	95% CI	5.98–10.67	0.61–1.05	60.89–70.48
Bound Phenolics					
*Cytospora* sp.	Amplitude 40%	Predicted	4.45	1.52	22.84
	Time 10 min	Experimental	4.49 ± 0.02	1.46 ± 0.04	20.15 ± 0.04
	NaOH 1.5 M	95% CI	4.21–4.67	1.42–1.61	21.60–23.90
*Durvilleae potatorum*	Amplitude 80%	Predicted	2.54	0.70	15.62
	Time 6 min	Experimental	2.97 ± 0.02	0.76 ± 0.02	14.51 ± 0.04
	NaOH 0.5 M	95% CI	2.31–2.98	0.67–0.74	14.8–16.4
*Sargassum fallax*	Amplitude 40%	Predicted	4.62	1.69	21.20
	Time 10 min	Experimental	4.34 ± 0.01	1.62 ± 0.01	19.96 ± 0.12
	NaOH 1.5 M	95% CI	4.39–8.65	1.61–1.77	19.84–22.26
*Ecklonia radiata*	Amplitude 40%	Predicted	1.42	0.38	4.26
	Time 8 min	Experimental	1.41 ± 0.00	0.48 ± 0.01	4.68 ± 0.05
	NaOH 1.5 M	95% CI	1.35–1.49	0.36–0.40	4.05–4.47
*Phyllospora comosa*	Amplitude 42%	Predicted	3.98	0.68	9.56
	Time 8 min	Experimental	4.24 ± 0.01	0.71 ± 0.01	9.91 ± 0.07
	NaOH 1.5 M	95% CI	3.78–4. 25	0.65–0.72	9.08–10.04

CI, Confidence Interval; GAE, gallic acid equivalent; PGE, phloroglucinol equivalent; TE, Trolox equivalent; TPC, total phenolic content; TPhC, total phlorotannin content; DPPH: 2,2-diphenyl-1-picrylhydrazyl radical scavenging activity.

**Table 5:** Free and bound phenolic contents of seaweed samples extracted using conventional and ultrasonication methods.

	Samples	TPC (mg GAE/g)	TFC (mg QE/g)	TCT (mg CE/g)	TPhC (mg PGE/g)	DPPH (mg TE/g)	ABTS (mg TE/g)	FRAP (mg TE/g)	PRAC (mg TE/g)	FICA (mg EDTA-E/g)
*Free* *Phenolics*	Conventional Extraction									
	*Cytospora* sp.	10.62 ± 0.24 ^Aa^	0.34 ± 0.02 ^Aa^	2.39 ± 0.51 ^Aa^	1.22 ± 0.04 ^Aa^	39.97 ± 0.64 ^Aa^	44.86 ± 1.03 ^Aa^	19.91 ± 0.35 ^Aa^	22.92 ± 0.53 ^Aa^	3.32 ± 0.14 ^Aa^
	*Durvillaea potatorum*	3.61 ± 0.01 ^Ab^	0.03 ± 0.00 ^Ab^	-	0.15 ± 0.00 ^Ab^	20.85 ± 0.26 ^Ab^	20.27 ± 0.19 ^Ab^	0.98 ± 0.04 ^Ab^	0.36 ± 0.02 ^Ab^	3.17 ± 0.11 ^Aab^
	*Sargassum fallax*	17.43 ± 0.02 ^Ac^	0.41 ± 0.02 _Ac_	-	0.74 ± 0.02 ^Ac^	49.97 ± 1.14 ^Ac^	50.40 ± 1.80 ^Ac^	24.52 ± 0.77 ^Ac^	46.37 ± 0.77 ^Ac^	3.05 ± 0.01 ^Ab^
	*Ecklonia radiata*	8.95 ± 0.06 ^Ad^	0.01 ± 0.00 ^Ab^	6.23 ± 0.61 ^Ab^	2.03 ± 0.03 ^Ad^	32.80 ± 0.37 ^Ad^	46.87 ± 0.79 ^Aa^	15.19 ± 0.43 ^Ad^	3.11 ± 0.08 ^Ad^	2.74 ± 0.04 ^Ac^
	*Phyllospora comosa*	4.03 ± 0.09 ^Ae^	0.03 ± 0.00 ^Ab^	-	0.35 ± 0.01 ^Ae^	22.58 ± 0.36 ^Ae^	27.98 ± 0.78 ^Ad^	5.43 ± 0.19 ^Ae^	3.12 ± 0.06 ^Ad^	3.15 ± 0.05 ^Aab^
	Ultrasonic Extraction									
	*Cytospora* sp.	14.64 ± 1.12 ^Ba^	0.96 ± 0.05 ^Ba^	4.35 ± 0.47 ^Ba^	2.42 ± 0.23 ^Ba^	60.67 ± 0.17 ^Ba^	88.08 ± 1.55 ^Ba^	29.21 ± 0.46 ^Ba^	39.60 ± 0.66 ^Ba^	11.78 ± 0.14 ^Ba^
	*Durvillaea potatorum*	4.01 ± 0.29 ^Ab^	0.43 ± 0.02 ^Bb^	3.48 ± 0.21 ^b^	2.26 ± 0.25 ^Bb^	35.26 ± 0.08 ^Bb^	31.44 ± 0.68 ^Bb^	1.17 ± 0.04 ^Bb^	0.84 ± 0.04 ^Bb^	7.52 ± 0.08 ^Bb^
	*Sargassum fallax*	20.32 ± 0.41 ^Bc^	1.28 ± 0.02 ^Bc^	-	1.16 ± 0.29 ^Ac^	73.66 ± 0.51 ^Bc^	86.88 ± 0.40 ^Ba^	33.70 ± 0.68 ^Bc^	60.68 ± 0.05 ^Bc^	6.76 ± 0.06 ^Bc^
	*Ecklonia radiata*	11.6 ± 0.57 ^Bd^	1.01 ± 0.02 ^Bd^	7.80 ± 0.18 ^Bc^	2.64 ± 0.15 ^Bb^	66.84 ± 0.34 ^Bd^	80.38 ± 0.39 ^Bc^	17.70 ± 0.56 ^Bd^	5.53 ± 0.21 ^Bd^	5.95 ± 0.17 ^Bd^
	*Phyllospora comosa*	8.15 ± 0.20 ^Be^	0.94 ± 0.01 ^Ba^	0.38 ± 0.12 ^d^	0.81 ± 0.04 ^Bc^	64.38 ± 0.80 ^Be^	39.95 ± 0.90 ^Bd^	9.60 ± 0.04 ^Be^	3.72 ± 0.12 ^Be^	5.42 ± 0.11 ^Be^
*Bound* *Phenolics*	Conventional Extraction									
	*Cytospora* sp.	1.25 ± 0.04 ^Aa^	0.10 ± 0.01 ^Aab^	-	0.18 ± 0.00 ^Aa^	3.77 ± 0.11 ^Aa^	7.63 ± 0.11 ^Aa^	3.20 ± 0.08 ^Aa^	3.51 ± 0.02 ^Aa^	1.00 ± 0.01 ^Aa^
	*Durvillaea potatorum*	2.40 ± 0.04 ^Ab^	0.01 ± 0.00 ^Ac^	5.54 ± 0.02 ^Aa^	0.42 ± 0.01 ^Ab^	5.18 ± 0.12 ^Ab^	8.71 ± 0.20 ^Ab^	2.09 ± 0.05 ^Ab^	0.21 ± 0.01 ^Ab^	0.60 ± 0.01 ^Ab^
	*Sargassum fallax*	0.66 ± 0.02 ^Ac^	0.13 ± 0.02 ^Ad^	-	0.08 ± 0.00 ^Ac^	2.97 ± 0.01 ^Ac^	4.00 ± 0.00 ^Ac^	2.07 ± 0.01 ^Ab^	0.43 ± 0.05 ^Ac^	0.56 ± 0.00 ^Ac^
	*Ecklonia radiata*	0.46 ± 0.01 ^Ad^	0.10 ± 0.01 ^Aa^	-	0.02 ± 0.00 ^Ad^	2.54 ± 0.05 ^Ad^	3.56 ± 0.06 ^Ad^	1.02 ± 0.04 ^Ac^	1.16 ± 0.01 ^Ad^	0.50 ± 0.01 ^Ad^
	*Phyllospora comosa*	0.30 ± 0.01 ^Ae^	0.10 ± 0.00 ^Ab^	0.19 ± 0.06 ^Ab^	0.15 ± 0.01 ^Ae^	1.83 ± 0.04 ^Ae^	2.75 ± 0.04 ^Ae^	1.32 ± 0.02 ^Ad^	1.25 ± 0.08 ^Ae^	0.46 ± 0.01 ^Ae^
	Ultrasonic Extraction									
	*Cytospora* sp.	4.49 ± 0.02 ^Ba^	0.25 ± 0.09 ^Ba^	-	1.46 ± 0.04 ^Ba^	20.15 ± 0.04 ^Ba^	21.42 ± 0.86 ^Ba^	8.44 ± 0.18 ^Ba^	10.4 ± 0.19 ^Ba^	1.42 ± 0.03 ^Ba^
	*Durvillaea potatorum*	2.97 ± 0.02 ^Bb^	0.07 ± 0.00 ^Bb^	6.91 ± 0.09 ^Aa^	0.76 ± 0.02 ^Bb^	14.51 ± 0.04 ^Bb^	17.96 ± 0.09 ^Bb^	2.25 ± 0.08 ^Bb^	6.15 ± 0.15 ^Bb^	0.92 ± 0.01 ^Bb^
	*Sargassum fallax*	4.34 ± 0.01 ^Bc^	0.00 ± 0.00 ^Bc^	1.63 ± 0.38 ^bc^	1.62 ± 0.01 ^Bc^	19.96 ± 0.12 ^Ba^	6.47 ± 0.18 ^Bc^	2.79 ± 0.01 ^Bc^	0.52 ± 2.62 ^Bc^	1.02 ± 0.03 ^Bc^
	*Ecklonia radiata*	1.41 ± 0.00 ^Bd^	0.08 ± 0.01 ^Bd^	3.15 ± 0.78 ^b^	0.48 ± 0.01 ^Bd^	4.68 ± 0.05 ^Bc^	5.65 ± 0.20 ^Bc^	1.16 ± 0.04 ^Bd^	2.12 ± 0.54 ^Bd^	1.10 ± 0.01 ^Bd^
	*Phyllospora comosa*	4.24 ± 0.01 ^Be^	0.04 ± 0.01 ^Be^	0.43 ± 0.08 ^Ac^	0.71 ± 0.01 ^Bb^	9.91 ± 0.07 ^Bd^	7.73 ± 0.34 ^Bd^	1.50 ± 0.03 ^Be^	1.95 ± 0.19 ^Bc^	1.01 ± 0.03 ^Bc^

The Note: The reported data for seaweeds are based on dry-weight measurements The data were presented as mean ± standard deviation (n = 3). ^a–e^ Different letter superscripts in the same column indicate a significant difference at *p* < 0.05 between different seaweed samples. ^A,B^ Different letter superscripts within a column for the same seaweed species indicate a significant difference at *p* < 0.05 between conventional and ultrasonic extraction. Abbreviations: ABTS (2,2′-azino-bis(3-ethylbenzothiazoline-6-sulfonic acid), CE (catechin equivalents), DPPH (2,2-diphenyl-1-picrylhydrazyl), EDTA-E (ethylenediaminetetraacetic acid equivalent), FICA (ferrous ion chelating activity), FRAP (ferric-reducing antioxidant power), GAE (gallic acid equivalents), PGE (phloroglucinol equivalents), PRAC (phosphomolybdate-reducing antioxidant capacity), QE (quercetin equivalents), TCT (total condensed tannin), TE (Trolox equivalents), TFC (total flavonoid content), TPC (total phenolic content), and TPhC (total phlorotannin content).

**Table 6:** Characterization of phenolic compounds extracted by conventional and ultrasonic methods using liquid chromatography electrospray ionization quadrupole time-of-flight mass spectrometry (LC–ESI-QTOF-MS/MS).

No.	Proposed Compounds	Molecular Formula	RT (min)	Ionization (ESI^+^/ESI^−^)	Molecular Weight	Theoretical (*m*/*z*)	Observed (*m*/*z*)	Error (ppm)	Product Ion (*m*/*z*)	Sample
PHENOLIC ACIDS										
Hydroxycinnamic acid										
1	1,2,2′-Triferuloylgentiobiose	C_42_H_46_O_20_	7.766	[M − H]^−^	870.2613	869.2540	869.2526	−1.6	693, 517	* CFEC, UFEC, CBEC
2	p-Coumaroyl malic acid	C_13_H_12_O_7_	64.185	[M − H]^−^	280.0575	279.0502	279.0509	2.5	163, 119	* UFCY, UBCY, CBDU, UFPH
3	1-Sinapoyl-2,2′-diferuloylgentiobiose	C_43_H_48_O_21_	67.219	[M − H]^−^	900.2671	899.2598	899.2617	2.1	613, 201	UFEC
Hydroxybenzoic acid										
4	3,4-*O*-Dimethylgallic acid	C_9_H_10_O_5_	40.416	[M + H]^+^	198.0537	199.061	199.061	0.0	153, 139, 125, 111	* CBDU, CBSA, CBPH, CFCY, CFPH, UBCY, UBDU, UBSA, UBPH
5	4-*O*-Methylgallic acid	C_8_H_8_O_5_	55.241	[M + H]^+^	184.0381	185.0454	185.0452	−1.1	170, 141	* CBSA, CFDU, UBCY, UBDU
FLAVONOIDS										
Flavanols										
6	Quercetin 3-*O*-xylosyl-glucuronide	C_26_H_26_O_17_	13.846	[M + H]^+^	610.1123	611.1196	611.1223	4.4	479, 303, 285, 239	* UFDU, CFDU, CFSA, UFSA
7	Quercetin 3′-sulfate	C_15_H_10_O_10_S	14.006	[M − H]^−^	381.9973	380.9900	380.9891	−2.4	301	* CBEC, CBDU
8	Prodelphinidin dimer B3	C_30_H_26_O_14_	54.815	[M + H]^+^	610.13	611.1373	611.1384	1.8	469, 311, 291	* UFCY, CFCY, UFPH
9	Spinacetin 3-*O*-glucosyl-(1->6)-glucoside	C_29_H_34_O_18_	62.464	[M − H]^−^	670.1743	669.1670	669.1655	−2.2	609, 301	CFDU
Anthocyanins										
10	Cyanidin 3-*O*-(6″-*p*-coumaroyl-glucoside)	C_30_H_27_O_13_	55.998	[M + H]^+^	595.1474	596.1547	596.1554	1.2	287	CFPH
11	Cyanidin 3-*O*-diglucoside-5-*O*-glucoside	C_33_H_41_O_21_	57.989	[M + H]^+^	773.2171	774.2244	774.2246	0.3	610, 464	UFCY
12	Peonidin 3-*O*-diglucoside-5-*O*-glucoside	C_34_H_43_O_21_	63.639	[M + H]^+^	787.2335	788.2408	788.2445	4.7	625, 478, 317	UBPH
Flavanones										
13	Neohesperidin	C_28_H_34_O_15_	13.784	[M + H]^+^	610.1908	611.1981	611.1985	0.7		CFPH
14	Hesperetin 5,7-*O*-diglucuronide	C_28_H_30_O_18_	63.386	[M − H]^−^	654.1372	653.1299	653.132	3.2	447, 301, 286, 242	* CFEC, CFDU
Flavones										
15	Nobiletin	C_21_H_22_O_8_	67.568	[M + H]^+^	402.1323	403.1396	403.1393	−0.7	359	* CFCY, UFCY
Isoflavonoids										
16	6″-*O*-Acetylglycitin	C_24_H_24_O_11_	13.841	** [M + H]^+^	488.1326	487.1253	487.1259	1.2	285, 270	* CFCY, CFSA, CFSA, CFPH, UFCY, UFSA, UFPH
OTHER POLYPHENOLS										
Hydroxycoumarins										
17	Coumarin	C_9_H_6_O_2_	13.576	[M + H]^+^	146.0379	147.0452	147.0451	−0.7	103, 91	UFCY
18	Esculin	C_15_H_16_O_9_	14.058	[M + H]^+^	340.0813	341.0886	341.0882	−1.2	179, 151	CBPH
Hydroxyphenylpropenes										
19	Eugenol	C_10_H_12_O_2_	65.708	[M + H]^+^	164.0845	165.0918	165.0916	−1.2	153	* CFPH, CBEC, UBCY, UBSA, UBEC, UBPH, UFPH
Hydroxybenzaldehydes										
20	*p*-Anisaldehyde	C_8_H_8_O_2_	63.263	[M + H]^+^	136.0528	137.0601	137.0602	0.7	122, 109	* CFEC, CBSA, CBEC, CFPH
Phenolic terpenes										
21	Epirosmanol	C_20_H_26_O_5_	59.444	[M + H]^+^	346.1793	347.1866	347.1852	−4.0	253	* CFEC, CBEC, CBPH, UBEC, UFEC
Curcuminoids										
22	Bisdemethoxycurcumin	C_19_H_16_O_4_	13.874	[M + H]^+^	308.1051	309.1124	309.1122	−0.6	291, 263	* CFDU, CFPH
LIGNANS										
23	Enterolactone	C_18_H_18_O_4_	65.845	[M + H]^+^	298.1184	299.1257	299.1257	0.0	281, 187, 165	CFEC
24	7-Oxomatairesinol	C_20_H_20_O_7_	57.618	[M + H]^+^	372.1212	373.1285	373.1276	−2.4	358, 343, 328, 325	CBPH
25	Schisandrin	C_24_H_32_O_7_	60.864	[M + H]^+^	432.2139	433.2212	433.2205	−1.6	415, 361	* CBEC, CBCY, CBPH

Note: RT, Retention Time; CF, Conventional Free; UF, Ultrasonication Free; CB, Conventional Bound; UB, Ultrasonication-Bound; CY, *Cytospora* sp.; DU, *Durvillaea* sp.; SA, *Sargassum* sp.; EC, *Ecklonia* sp.; PH, *Phyllospora* sp. * Compound was detected in more than one seaweed sample; data presented in this table are from the asterisk sample. ** Compounds were detected in negative [M − H]^−^ and positive [M + H]^+^ ion.

## Data Availability

The data presented in this study are available in this article.

## Supplementary Materials

The following supporting information can be downloaded at: https://www.mdpi.com/article/10.3390/antiox13040409/s1. Table S1. Experimental design for free phenolic extractions; Table S2. Experimental design for bound phenolic extractions; Table S3. Determination of levels of extraction variable for free phenolics; Table S4. Determination of levels of extraction variable for bound phenolics; Table S5. Regression equation and correlation coefficient of reference phenolic compounds studied.
