# Supplementary material for: Optimization of Ultrasonic Extraction Parameters for the Recovery of Phenolic Compounds in Brown Seaweed: Comparison with Conventional Techniques
Source: Antioxidants (Basel). 2024 Mar 28;13(4):409. doi: 10.3390/antiox13040409 (PMC11047748; doi:10.3390/antiox13040409)
Supplement: Supplementary file 1 [file antioxidants-13-00409-s001.zip › antioxidants-2925285-supplementary.pdf]

antioxidants

MDPI

Table S1. Experimental design for free phenolic extractions.

RunOrder	Amplitude (%)	Time (min)	Solvent : solid ratio
**1**	40	8	15
**2**	80	6	10
**3**	80	4	15
**4**	40	6	20
**5**	60	8	20
**6**	60	6	15
**7**	80	6	20
**8**	60	6	15
**9**	60	8	10
**10**	60	6	15
**11**	40	4	15
**12**	60	6	15
**13**	60	4	20
**14**	60	4	10
**15**	80	8	15
**16**	60	6	15
**17**	40	6	10

Table S2. Experimental design for bound phenolic extractions.

RunOrder	Amplitude (%)	Time (min)	NaOH concentration (M)
**1**	40	6	1.0
**2**	60	8	1.0
**3**	60	8	1.0
**4**	60	6	0.5
**5**	60	8	1.0
**6**	60	6	1.5
**7**	60	10	0.5
**8**	80	10	1.0
**9**	80	8	1.5
**10**	60	10	1.5
**11**	80	6	1.0
**12**	60	8	1.0
**13**	40	8	0.5
**14**	40	10	1.0
**15**	60	8	1.0
**16**	80	8	0.5
**17**	40	8	1.5

Table S3. Determination of levels of extraction variable for free phenolics.

Amplitude (%)	Time (mins)	Solvent-solid ratio	TPC (mg GAE/g)	TPhC (mg PGE/g)	DPPH (mg TE/g)
**20**	4	10:1	16.83 ± 1.46	1.32 ± 0.09	39.14 ± 1.58
40			18.08 ± 1.00	1.56 ± 0.13	50.43 ± 2.50
60			19.68 ± 1.13	1.64 ± 0.09	47.07 ± 0.74
80			23.16 ± 1.15	1.16 ± 0.8	58.11 ± 2.24
100			20.15 ± 1.39	1.33 ± 0.10	43.16 ± 0.47
40	2		15.58 ± 0.99	0.41 ± 0.02	36.96 ± 0.84
	6		18.36 ± 0.10	1.16 ± 0.01	45.95 ± 2.68
	8		20.58 ± 1.34	1.45 ± 0.11	51.72 ± 0.46
	10		23.22 ± 1.63	1.44 ± 0.09	38.65 ± 1.95
	4	15:1	25.08 ± 1.69	1.79 ± 0.06	88.49 ± 0.27
		20:1	17.5 ± 0.37	1.00 ± 0.08	56.07 ± 0.40
		25:1	16.28 ± 0.84	0.25 ± 0.01	94.61 ± 4.21
		30:1	14.7 ±1.04	1.63 ± 0.05	96.16 ± 2.26

Table S4. Determination of levels of extraction variable for bound phenolics.

Amplitude (%)	Time (mins)	NaOH concentration	TPC (mg GAE/g)	TPhC (mg PGE/g)	DPPH (mg TE/g)
20	4	2.0	5.88 ± 0.21	0.48 ± 0.01	7.33 ± 0.18
40			5.91 ± 0.02	0.61 ± 0.02	7.20 ± 0.12
60			6.39 ± 0.32	0.62 ± 0.02	7.40 ± 0.22
80			6.53 ± 0.18	0.65 ± 0.02	7.81 ± 0.27
100			6.41 ± 0.02	0.53 ± 0.02	7.67 ± 0.26
40	6		6.66 ± 0.41	0.70 ± 0.01	7.48 ± 0.33
	8		6.73 ± 0.41	0.85 ± 0.02	7.06 ± 0.08
	10		6.20 ± 0.63	0.69 ± 0.06	6.69 ± 0.07
	12		6.09 ± 0.24	0.66 ± 0.01	6.81 ± 0.02
	4	0.5	7.01 ± 0.02	0.79 ± 0.01	8.05 ± 0.50
		1.0	7.22 ± 0.02	0.93 ± 0.02	8.70 ± 0.02
		1.5	6.00 ± 0.17	0.69 ± 0.06	7.39 ± 0.15
		2.0	5.91 ± 0.02	0.61 ± 0.02	7.20 ± 0.12

Table S5. Regression equation and correlation coefficient of reference phenolic compounds studied.

Compound name	RT (min)	Wavelength (nm)	Regression Equation	Correlation coefficient (r)
**Phloroglucinol**	17.576	254	y = 1.7721x - 5.7251	0.994
**Gallic acid**	21.37	280	y = 2.2745x - 6.333	0.980
**4-hydroxybenzoic acid**	41.844	254	y = 62.27x + 131.23	0.991
**Catechin**	49.769	254	y = 41.401x + 56.597	0.992
**Chlorogenic acid**	52.424	320	y = 13.421x - 13.635	0.997
**Syringic acid**	55.544	254	y = 15.567x + 21.217	0.998
**Epicatechin**	61.998	254	y = 1.2394x - 0.4619	0.990
**Epigallocatechin**	64.629	280	y = 7.313x + 3.3	0.998
**Sinapic acid**	79.016	320	y = 29.514x - 51.96	0.984

* RT: retention time.
